# Brain transforms natural killer cells that exacerbate brain edema after intracerebral hemorrhage

**DOI:** 10.1084/jem.20200213

**Published:** 2020-09-01

**Authors:** Zhiguo Li, Minshu Li, Samuel X. Shi, Nan Yao, Xiaojing Cheng, Ai Guo, Zilong Zhu, Xiaoan Zhang, Qiang Liu

**Affiliations:** 1 Department of Neurology, Tianjin Neurological Institute, Tianjin Medical University General Hospital, Tianjin, China; 2 National Clinical Research Center for Neurological Disease of China, Jing-Jin Center for Neuroinflammation, Beijing Tiantan Hospital, Capital Medical University, Beijing, China; 3 Interdisciplinary Neuroscience Graduate Program, Arizona State University, Tempe, AZ; 4 Department of Neurology, Tianjin Huanhu Hospital, Tianjin, China; 5 Department of Radiology, The Third Affiliated Hospital of Zhengzhou University, Zhengzhou, Henan, China

## Abstract

Perihematomal edema (PHE) occurs within hours after intracerebral hemorrhage (ICH), leading to secondary injury manifested by impaired blood–brain barrier (BBB) integrity and destruction of adjacent tissue. To dissect the mechanisms underlying PHE formation, we profiled human and mouse perihematomal tissues and identified natural killer (NK) cells as the predominant immune cell subset that outnumbers other infiltrating immune cell types during early stages of ICH. Unbiased clustering of single-cell transcriptional profiles revealed two major NK cell subsets that respectively possess high cytotoxicity or robust chemokine production features in the brain after ICH, distinguishing them from NK cells of the periphery. NK cells exacerbate BBB disruption and brain edema after ICH via cytotoxicity toward cerebral endothelial cells and recruitment of neutrophils that augment focal inflammation. Thus, brain-bound NK cells acquire new features that contribute to PHE formation and neurological deterioration following ICH.

## Teaser

Intracerebral hemorrhage (ICH) provokes brain edema and causes high mortality and disability. This study has identified brain-bound natural killer cells as a key contributor to brain edema and neurological deterioration after ICH.

## Graphical Abstract

**Figure:**
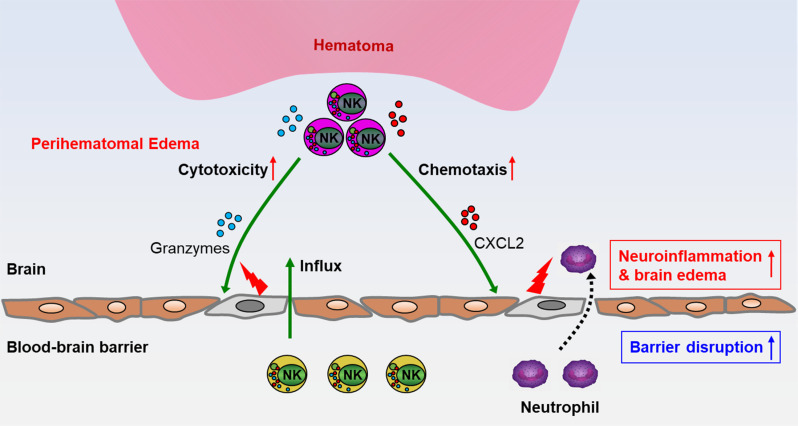

## Introduction

Intracerebral hemorrhage (ICH) accounts for 10–15% of all strokes and is associated with high mortality and morbidity. ICH not only causes primary brain injury through direct mechanical effects of the hematoma, but also leads to the development of perihematomal edema (PHE), which induces secondary brain injury manifested by impaired blood–brain barrier (BBB) integrity and adjacent tissue destruction (Aronowski and Zhao, 2011; Keep et al., 2012; Urday et al., 2015). PHE occurs early after ICH, with a sharp increase ∼75% of its maximum volume within the first day, and continues to develop over an extended time of days to weeks thereafter (Qureshi et al., 2009; Urday et al., 2015). Preclinical studies indicate that PHE augments mass effects caused by the initial hematoma and imposes direct damage to cerebral tissue via dysregulation of osmotic gradients and facilitation of barrier disruption, leading to neuronal loss and long-term disability (Lee et al., 1997; Thiex and Tsirka, 2007; Urday et al., 2015). Clinically, the extent of PHE is associated strongly with poor outcome in ICH patients (Murthy et al., 2016; Urday et al., 2015, 2016). However, clinical trials of targeting ICH hematoma by surgical evacuation or endoscopic clot aspiration with tissue plasminogen activator have not demonstrated therapeutic efficacy (Hanley et al., 2019). Similarly, the effectiveness of pharmacological interventions such as hyperosmolar therapy and iron chelation either is uncertain or awaits further investigation in ICH patients (Selim et al., 2019; Urday et al., 2015). As such, ICH remains the least treatable form of stroke. Considering the contribution of PHE to secondary clinical deterioration and mortality, PHE may represent an attractive therapeutic target in ICH.

ICH results in a rapid and robust cellular immune response characterized in part by activation of neuroglia and infiltration of leukocytes that release proinflammatory factors (Fu et al., 2015; Murthy et al., 2016; Urday et al., 2015). Evidence indicates that inflammation precipitated by leukocytes homing into the brain and blood components released from the clot accelerates PHE formation, exacerbates mass effect, and amplifies cell death (Fu et al., 2015; Iadecola and Anrather, 2011; Keep et al., 2012). Therefore, focal inflammation contributes significantly to BBB breakdown and brain edema. Conversely, BBB disruption also promotes inflammation by allowing the infiltration of leukocytes, which exacerbates brain edema after ICH. Among the major leukocyte subsets, lymphocytes are found in cerebrospinal fluid as early as 6 h after ICH, as well as in perihematomal brain tissue obtained from ICH patients (Fu et al., 2015; Mracsko and Veltkamp, 2014). Moreover, previous studies report the detrimental effects of myeloid cells such as neutrophils and monocytes in ICH (Aronowski and Zhao, 2011; Mracsko and Veltkamp, 2014). However, whether and how lymphocytes contribute to acute brain edema and control migration of these myeloid cells remain elusive.

Natural killer (NK) cells are large granular lymphocytes that constitute the third lymphocyte population, along with T and B cells (Vivier et al., 2011). NK cells rapidly respond to sterile stimulus-like alarmins and chemokines released by the injured brain (Kaur et al., 2013; Shi et al., 2011). Once activated, NK cells possess cytotoxic activity and produce cytokines and chemokines, by which they orchestrate other immune cells to limit or intensify immune responses (Long et al., 2013; Shi et al., 2011; Vivier et al., 2011). Particularly, through cooperation with myeloid cells, NK cells can facilitate their production of inflammatory cytokines to amplify local immune response (Vivier et al., 2011). Given the prompt nature of NK cells and the rapid PHE expansion after acute ICH, we postulated that NK cells aggravate PHE expansion and ICH injury via cytotoxic activity and magnification of local inflammation. In this study, we found that NK cells swiftly arrive in the brain after ICH, augment focal inflammation, and contribute to early PHE formation and neurological deterioration.

## Results

### Differential features of NK cells in brain and periphery after ICH in humans and mice

**Figure 1.**
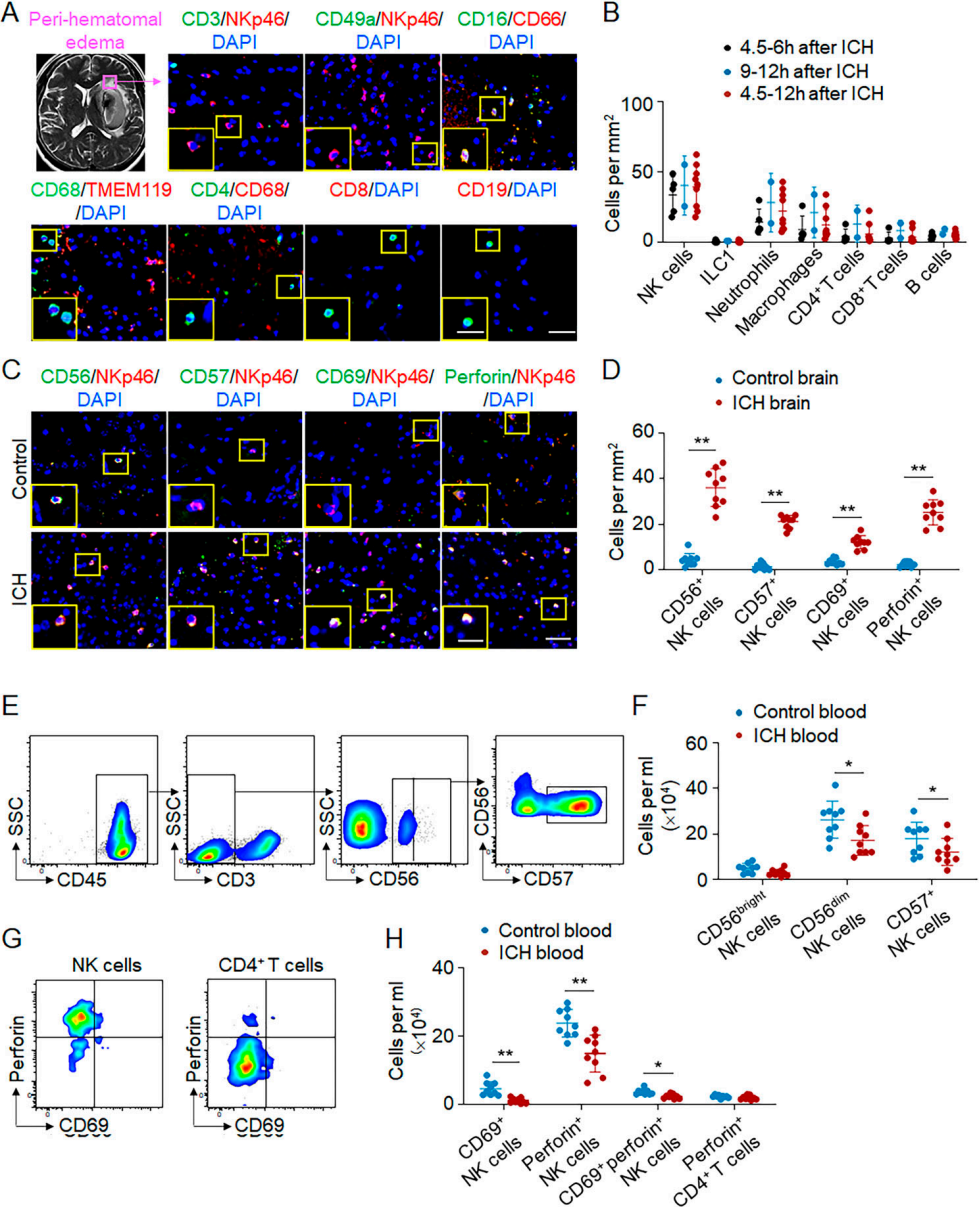
**NK cell invasion into the perihematomal brain tissues of ICH patients.** Perihematomal brain tissues were obtained from ICH patients who underwent urgent evacuation of hematoma within 12 h after ictus. Blood samples were collected from healthy individuals or ICH patients within 24 h after ictus. **(A)** T2/Flair MR image showing PHE region in the brain of an ICH patient. Immunostaining of brain sections containing perihematomal tissues revealed NK cells (CD3^−^NKp46^+^), ILC1 (CD49a^+^NKp46^+^), neutrophils (CD16^+^CD66^+^), macrophages (CD68^+^TMEM119^−^), CD4^+^ T cells (CD4^+^CD68^−^), CD8^+^ T cells (CD8^+^), and B cells (CD19^+^). Scale bars: 50 µm; 20 µm (inset). **(B)** Quantification of indicated immune cell subsets within PHE region of brain sections from ICH patients. 4.5–6-h group, *n* = 5 cases; 9–12-h group, *n* = 2 cases; 4.5–12-h group, *n* = 9 cases. Data are from three independent experiments. **(C and D)** Immunostaining (C) and quantification (D) of CD56^+^NKp46^+^ cells, CD57^+^NKp46^+^ cells, CD69^+^NKp46^+^ cells, and perforin^+^NKp46^+^ cells in perihematomal region within 12 h after ICH or controls. Postmortem brain tissues from patients without history of neurological diseases were used as controls. Scale bars: 50 µm; 20 µm (inset). *n* = 9/group. Data are from three independent experiments. **, P < 0.01. Unpaired two-tailed *t* test. **(E and F)** Flow cytometry plots (E) and quantification (F) showing CD56^bright^ NK cells, CD56^dim^ NK cells, and CD57^+^ NK cells in blood from ICH patients within 24 h of onset and healthy controls. *n* = 9/group. Data are from three independent experiments. *, P < 0.05. Unpaired two-tailed *t* test. **(G and H)** Flow cytometry plots (G) and quantification (H) showing CD69^+^ NK cells and perforin^+^ NK cells in blood from ICH patients 24 h after onset and healthy controls. All gates were set using fluorescence minus one (FMO) controls. CD4^+^ T cells were used as a control for perforin measurement. *n* = 9/group. Data are from three independent experiments. *, P < 0.05; **, P < 0.01. Unpaired two-tailed *t* test. Data are presented as mean ± SD.

PHE sharply increases within the first 24 h after ICH onset. To address the potential contribution of focal inflammation to the early expansion of PHE, we quantified the number of immune cell subsets within the perihematomal tissues obtained from ICH patients subjected to surgical evacuation of hematoma within 12 h after ictus. Immunostaining revealed that the number of NK cells within perihematomal region was greater than that of other infiltrating immune cell subsets including neutrophils, macrophages, CD4^+^ T cells, and CD8^+^ T and B cells (Fig. 1, A and B). These infiltrating NK cells express activation marker CD69 and cytotoxicity marker perforin (Fig. 1, C and D). We also found increased counts of CD56^+^ or CD57^+^ NK cells in brain sections from ICH patients (Fig. 1, C and D). Conversely, NK cells and their expression of CD69 or perforin were sparsely detected in brain tissues from control subjects without a history of neurological diseases (Fig. 1, C and D). In contrast to the increase of NK cells within the ICH brain, a reduced number of NK cells and expression of CD69 and perforin were observed in the circulation after ICH onset, together with reduced counts of circulating CD56^dim^ or CD57^+^ NK cells (Fig. 1, E and F).

**Figure 2.**
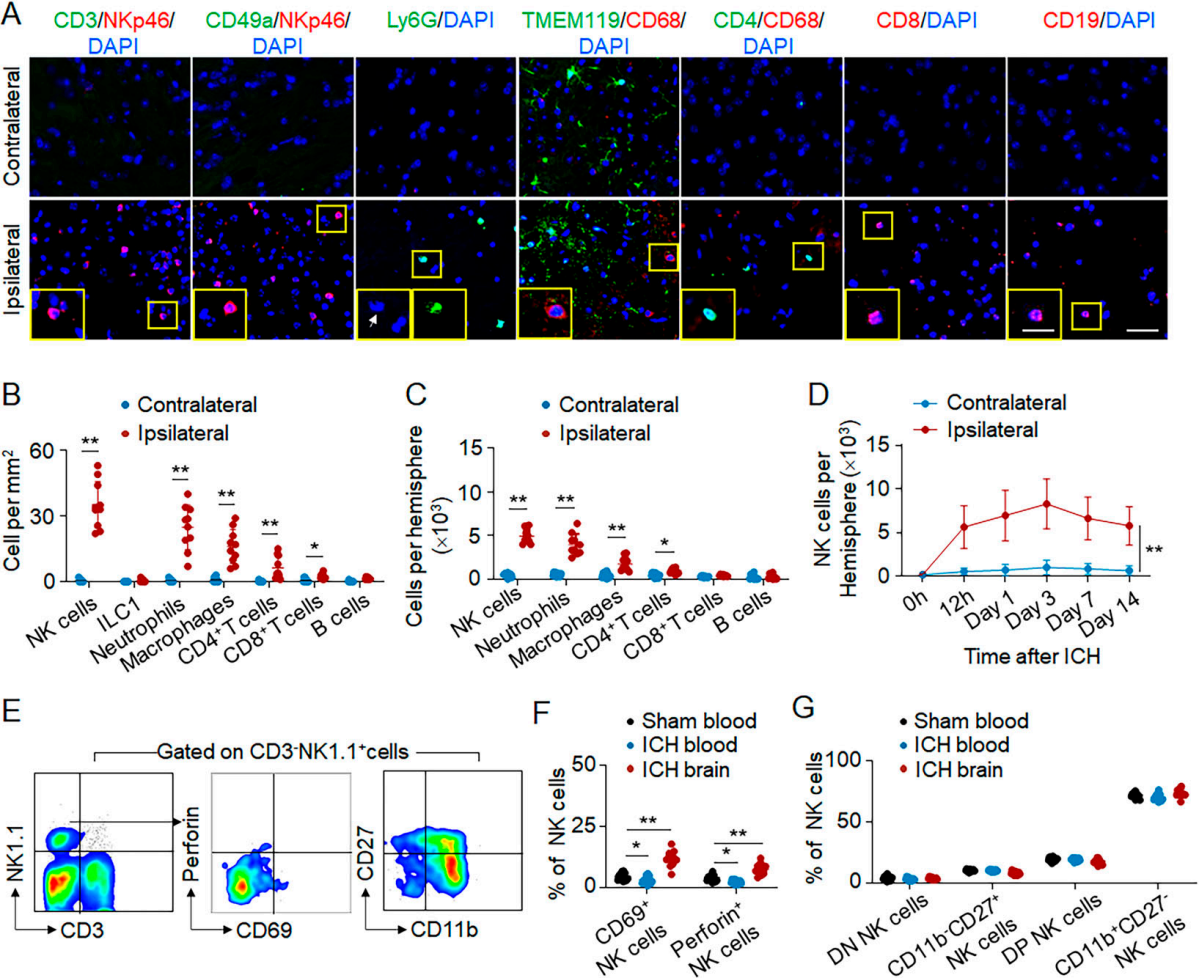
**Accumulation and activation of NK cells in the brain of ICH mice.** ICH was induced by autologous blood injection in C57BL/6 mice. 12 h after ICH, brain tissue and blood were isolated for flow cytometry analysis or immunostaining. **(A and B)** Immunostaining (A) and quantification (B) of immune cell subsets in the perihematomal tissues of ICH mice. *n* = 10/group. Scale bars: 50 µm; 20 µm (inset). Data are from three independent experiments. *, P < 0.05; **, P < 0.01. Unpaired two-tailed *t* test. **(C)** Counts of the different immune cell subsets in the ipsilateral hemisphere without hematoma or contralateral hemisphere 12 h after ICH. *n* = 10/group. Data are from three independent experiments. *, P < 0.05; **, P < 0.01. Unpaired two-tailed *t* test. **(D)** The dynamics of brain-infiltrating NK cells at indicated time points after ICH. *n* = 10/group. Data are from three independent experiments. **, P < 0.01. Two-way ANOVA. **(E and F)** Flow cytometry plots (E) and quantification (F) show the expression of CD69 and perforin in NK cells obtained from blood and brain tissues of ICH mice. Gates were set using fluorescence minus one (FMO) controls. *n* = 12/group. Data are from three independent experiments. *, P < 0.05; **, P < 0.01. One-way ANOVA. **(G)** CD11b and CD27 expression in NK cells obtained from blood and brain tissues in sham or ICH mice 12 h after surgery. DN, double negative; DP, double positive. *n* = 12/group. Data are from three independent experiments. Data are presented as mean ± SD.

**Figure S1.**
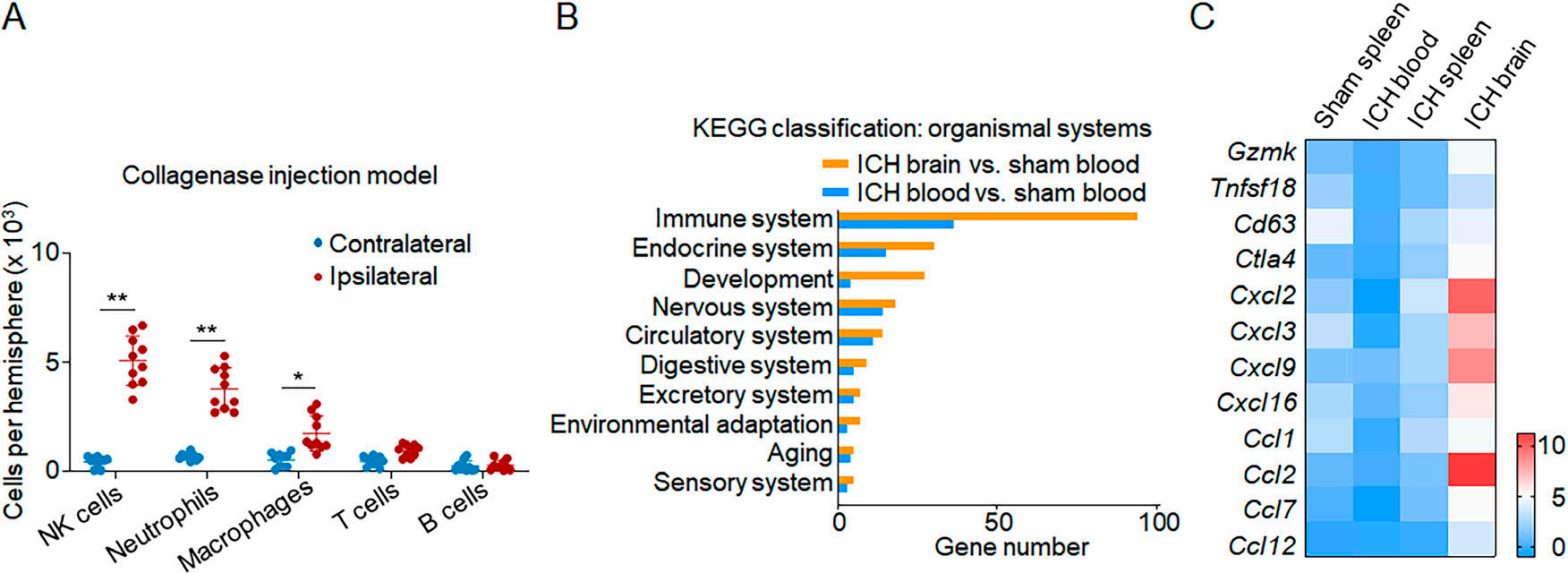
**Enrichment of immune signatures in brain-infiltrating NK cells following ICH. (A)** ICH was induced in C57BL/6 mice by collagenase injection. Quantification showing flow cytometry analysis of brain-infiltrating immune cell subsets including NK cells 12 h after ICH. *n* = 10/group. Data are from three independently repeated experiments. *, P < 0.05; **, P < 0.01. Unpaired two-tailed *t* test. **(B)** ICH was induced in C57BL/6 mice by autologous blood injection. Sham mice were used as controls. 12 h after ICH, NK cells were isolated from blood and brain tissues. The diversity of NK cells was assessed by single-cell RNA-seq on individual cells isolated from blood and brain. Enrichment of DEGs in NK cells classified by organ systems. Gene expression levels in NK cells from ICH brain and ICH blood were normalized to NK cells from sham blood. Data are from two independently repeated experiments. **(C)** ICH was induced in C57BL/6 mice by autologous blood injection. RT-PCR analysis was performed to measure an array of NK cell–related cytotoxicity- and chemokine-related genes. NK cells were sorted from spleen, blood, and brain tissues of indicated groups of sham or ICH mice. NK cells from blood and spleen of sham mice were used as controls. Heatmap showing the expression of cytotoxicity- and chemokine-related genes in indicated groups of NK cells. Data are from three independently repeated experiments. Data are shown as the fold change of genes versus sham blood group.

To recapitulate the dynamic expansion of PHE observed after clinical ICH, we used a mouse model of ICH induced by injection of autologous blood. Correspondingly, immunostaining revealed dramatic infiltration of NK cells within PHE regions, exceeding other recruited immune cell subsets 12 h after ICH onset (Fig. 2, A and B). Similarly, we also found an increase of infiltrating NK cells in the perihematomal regions using another mouse model of ICH induced by injection of collagenase at 12 h after ICH (Fig. S1 A).

Flow cytometry analysis revealed that these infiltrating NK cells accumulate within the ipsilateral hemisphere (Fig. 2 C) and peaked within 3 d after ICH (Fig. 2 D). Up-regulation of CD69 and perforin was seen in brain-infiltrating NK cells compared with circulating NK cells (Fig. 2, E and F). In addition, brain-infiltrating NK cells are mainly CD11b^+^CD27^+^ or CD11b^+^CD27^−^ subsets (Fig. 2 G). Together, these results demonstrate alterations of brain-infiltrating NK cells after ICH in humans and mice, indicating that NK cells swiftly respond to ICH and infiltrate into PHE tissues after ictus.

### Brain shapes NK cell features after ICH

**Figure 3.**
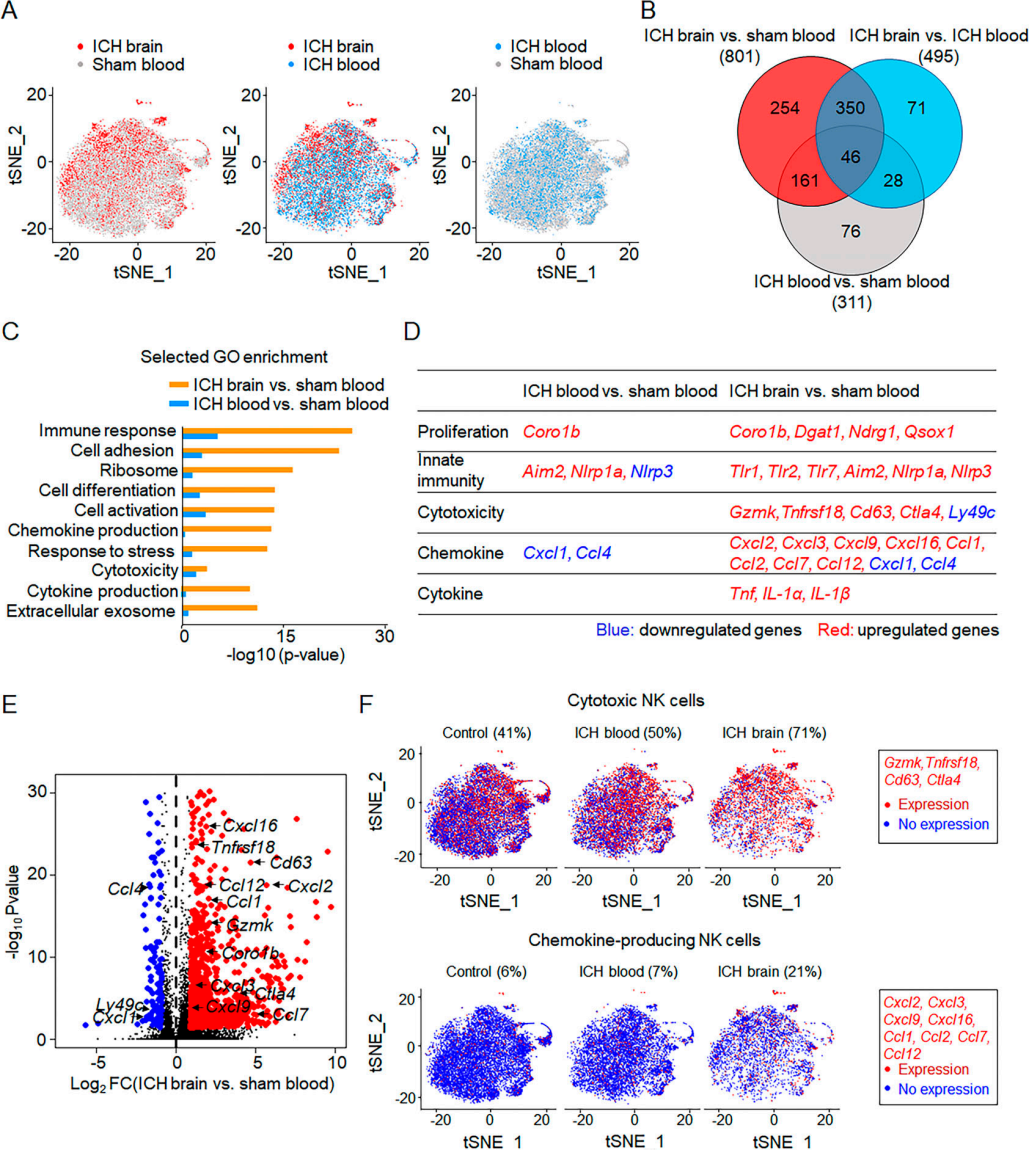
**Brain alters transcriptome profiles of infiltrating NK cells after ICH.** ICH was induced by autologous blood injection in C57BL/6 mice. Sham mice were used as controls. 12 h after ICH, NK cells were isolated from blood and brain tissues. The diversity of NK cells was assessed by single-cell RNA-seq on individual cells isolated from blood and brain. **(A)** t-SNE plots of NK cells obtained from ICH brain (*n* = 4,065 cells), ICH blood (*n* = 8,506 cells), and sham blood (control, *n* = 12,070 cells). NK cells were defined as CD45^+^CD3^−^NK1.1^+^NKp46^+^CD200R1^−^CD49a^−^. Data are from two independently repeated experiments. **(B)** Venn diagram of the DEGs between NK cells from ICH brain, ICH blood, and sham blood. The numbers indicate the unique and common DEGs in different comparisons. Data are from two independently repeated experiments. **(C)** Selected gene ontology enrichment of genes expressed in NK cells obtained from ICH brain and ICH blood versus NK cells from sham blood. Data are from two independently repeated experiments. **(D)** DEGs encoding cell proliferation, cytotoxicity, chemokine production, and cytokine production in NK cells from ICH brain and ICH blood versus NK cells from sham blood. Data are from two independently repeated experiments. **(E)** Volcano plot showing the fold change of genes (log_2_ scale) of NK cells in ICH brain compared with NK cells in sham blood (x axis) and their significance (y axis, −log_10_P value). Highly significant genes are indicated by a red dot (up-regulated) or blue dot (down-regulated), and genes related to cytotoxicity and chemokines are labeled on the plot. Data are from two independently repeated experiments. **(F)** t-SNE plots show brain-infiltrating NK cells with higher cytotoxicity or increased chemokine production after ICH versus NK cells from ICH blood or sham blood. Cytotoxicity or chemokine genes expressed by NK cells are in red. Data are from two independently repeated experiments.

We performed single-cell RNA sequencing (RNA-seq) in individual cells obtained from mouse brain and blood 12 h after ICH. Unbiased transcriptional clustering revealed alterations in gene expression pattern of brain-infiltrating NK cells obtained from ICH mice versus circulating NK cells obtained from either ICH mice or sham controls (Fig. 3 A). Among brain-infiltrating CD3^−^NK1.1^+^NKp46^+^ cells, 4.8% of these cells express group 1 innate lymphoid cell (ILC1) markers such as CD200R1 and CD49a. Among total CD3^−^NK1.1^+^NKp46^+^ cells in sham blood and ICH blood, the frequency of ILC1 was 3% and 2.7%, respectively. These results suggest that brain-infiltrating CD3^−^NK1.1^+^NKp46^+^ cells are predominantly NK cells (CD45^+^CD3^−^NK1.1^+^NKp46^+^CD49a^−^CD200R1^−^) but not ILC1. We identified 801 differentially expressed genes (DEGs) in NK cells obtained from ICH brain and 311 DEGs in NK cells obtained from ICH blood compared with sham controls (Fig. 3 B). These DEGs were most significantly enriched in the immune system, as defined by Kyoto Encyclopedia of Genes and Genomes classification (Fig. S1 B). Owing to the lack of NK cells in the sham brain tissues, we compared NK cells in the ICH brain versus sham blood or ICH blood. Gene ontology enrichment analysis revealed that brain-infiltrating NK cells displayed features of increased cell activation, cytotoxicity, and production of cytokines and chemokines after ICH (Fig. 3 C). Most genes related to cell proliferation, immune response, cytotoxicity, and production of cytokines and chemokines were up-regulated in brain-infiltrating NK cells but not circulating NK cells after ICH (Fig. 3, D and E). We mapped individual cells to two dimensions using t-distributed stochastic neighbor embedding (t-SNE) to visualize cell clustering based on their transcriptomic profiles. Notably, we identified increased expression of cytotoxic genes and chemokine genes in NK cells within the ICH brain (Fig. 3 F). In particular, the up-regulation of specific cytotoxic genes (*Gzmk*, *Tnfrsf18*, *Cd63*, and *Ctla4*) and chemokine genes (*Cxcl2*, *Cxcl3*, *Cxcl9*, *Cxcl16*, *Ccl1*, *Ccl2*, *Ccl7*, and *Ccl12*) were observed in brain-infiltrating NK cells (Fig. 3 F).

In addition, we measured an array of NK cell–related genes using RT-PCR in NK cells obtained from spleen, blood, and brain of ICH mice. NK cells from blood and spleen of sham mice were used as controls. Consistent with previous reports of NK cells in the spleen versus blood (Crinier et al., 2018), we found a mild increase of genes related to NK cell activity in the spleen versus blood of sham mice (Fig. S1 C). In the setting of ICH, brain NK cells expressed higher levels of genes related to cytotoxicity or chemokines than NK cells from spleen or blood (Fig. S1 C).

Together, these results revealed altered NK cell transcriptional profiles within the brain, indicating the increase of cytotoxicity and chemokine production in NK cells after homing into the ICH brain.

### NK cells exacerbate brain injury in ICH mice

**Figure S2.**
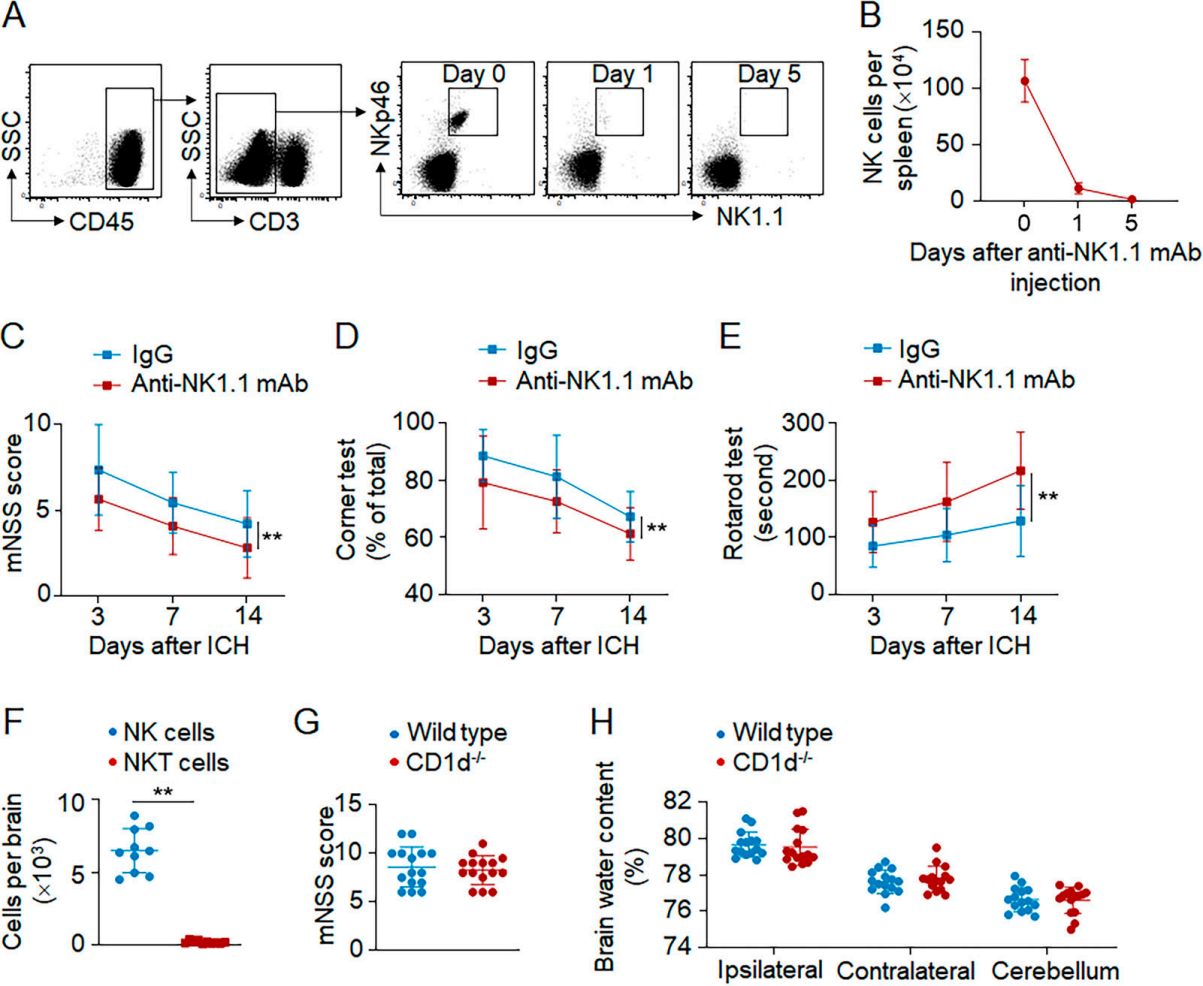
**In vivo NK cell depletion using an anti-NK1.1 mAb. (A and B)** ICH was induced in C57BL/6 mice by autologous blood injection. Mice received i.p. injection of antiNK1.1 mAb or IgG control 24 h before ICH surgery. For each mouse, 200 µg anti-NK1.1 mAb was injected every 5 d until the end of experiments. Spleen tissues were collected on days 0, 1, and 5 after anti-NK1.1 mAb injection. Flow cytometry (A) and quantification (B) of NK cells in spleen tissues of mice receiving anti-NK 1.1 mAb or IgG control. *n* = 6/group. Data are from three independently repeated experiments. **(C–E)** Mice received i.p. injection of anti-NK1.1 mAb or IgG control 24 h before ICH induction by collagenase injection. Neurological deficits were evaluated at indicated time points in ICH mice receiving anti-NK1.1 mAb or IgG. *n* = 15 mice/group. Data are from three independently repeated experiments. **, P < 0.01. Two-way ANOVA. **(F)** ICH was induced by autologous blood injection in wild-type C57BL/6 mice. Flow cytometry plots show minimal amount of brain-infiltrating NKT cells compared with brain-infiltrating NK cells 24 h after ICH. *n* = 10 mice/group. Data are from three independently repeated experiments. **, P < 0.01. Unpaired two-tailed *t* test. **(G and H)** ICH was induced by autologous blood injection in CD1d^−/−^ and wild-type C57BL/6 mice. Neurological deficits (G) and brain water content (H) were assessed on day 3 after ICH in indicated groups of mice. *n* = 15/group. Data are from three independently repeated experiments. Data are presented as mean ± SD.

**Figure 4.**
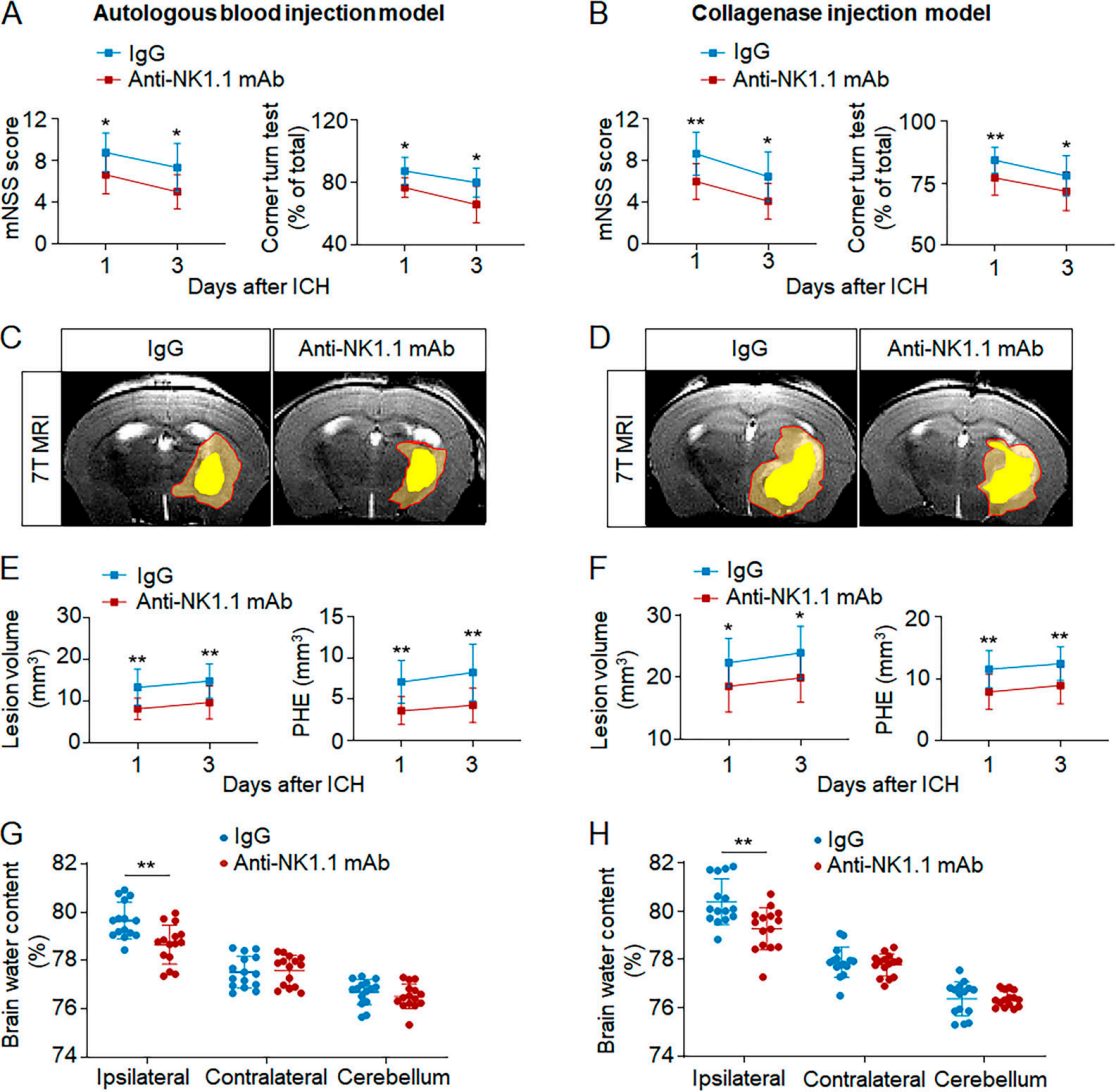
**NK cell depletion attenuates neurological deficits and brain edema in two mouse models of ICH.** ICH was induced by injection of autologous blood or collagenase in C57BL/6 mice. NK cells were depleted using anti-NK1.1 mAb 1 d before ICH induction. Brain water content was evaluated 1 d after ICH. Clinical assessment and PHE volume were evaluated on days 1 and 3 after ICH. **(A and B)** Effects of NK cell depletion on neurological deficits in ICH mice induced by injection of blood (A) or collagenase (B) at indicated time points after ICH. *n* = 15/group. Data are from three independent experiments. *, P < 0.05; **, P < 0.01. Two-way ANOVA. **(C–F)** 7T-MR images and quantification of lesion volume and PHE volume in ICH mice receiving anti-NK 1.1 mAb versus IgG controls. Multimodal 7T MRI was performed to visualize lesion (T2) and hematoma (SWI). PHE volume was calculated by subtracting the hematoma volume from total lesion volume. Red lines delineate lesion area, and yellow shaded regions represent hematoma area. *n* = 15/group. Data are from three independent experiments. *, P < 0.05; **, P < 0.01. Two-way ANOVA. **(G and H)** NK cell depletion reduced brain water content at indicated time points after ICH induced by injection of blood (G) or collagenase (H). Brain water content was measured 1 d after ICH. *n* = 15/group. Data are from three independent experiments. **, P < 0.01. Unpaired two-tailed *t* test. Data are presented as mean ± SD.

**Figure S3.**
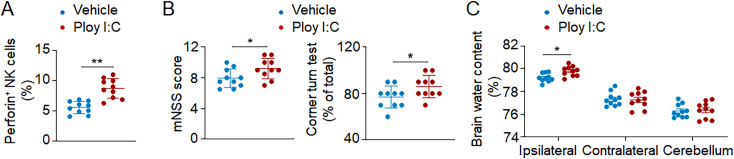
**Poly I:C stimulation of NK cells exacerbates ICH injury. (A–C)** NK cells were sorted from spleen tissues of wild-type C57BL/6 mice and cultured with vehicle or Poly I:C for 12 h in vitro. ICH was induced by autologous blood injection in Rag2^−/−^γc^−/−^ mice (devoid of T, B, and NK cells). Thereafter, Rag2^−/−^γc^−/−^ mice received adoptive transfer of NK cells that were treated with vehicle or Poly I:C. Quantification showing brain-infiltrating NK cells expressing perforin (A), neurological deficits (mNSS score and corner turn test assessment; B), and brain water content (C) in indicated groups of Rag2^−/−^γc^−/−^ mice 24 h after ICH. *n* = 10/group. Data are from three independently repeated experiments. *, P < 0.05; **, P < 0.01. Unpaired two-tailed *t* test. Data are presented as mean ± SD.

To understand whether NK cells contribute to ICH injury, we used anti-NK1.1 mAb (PK136) to deplete NK cells (Li et al., 2017b; Liu et al., 2016, 2017). Mice received anti-NK1.1 mAb injection 24 h before ICH surgery. Anti-NK1.1 mAb was injected every 5 d until the end of experiments, and NK cells were efficiently depleted (Fig. S2, A and B). We found significantly reduced neurodeficits, PHE volume, and brain edema in ICH mice receiving anti-NK1.1 mAb (Fig. 4, A–H). ICH mice receiving anti-NK1.1 mAb had improved neurological outcome until 14 d after induction (Fig. S2, C–E). Anti-NK1.1 mAb depletes both NK cells and NKT cells, so we compared ICH injury in CD1d^−/−^ mice (devoid of NKT cells) and wild-type mice (with NKT cells). Similar neurological deficits, PHE volumes, and brain water content were observed in CD1d^−/−^ mice and wild-type mice after ICH (Fig. S2, F–H), indicating that NKT cells do not discernably influence ICH injury. Additionally, we stimulated cultured NK cells with Poly I:C and then injected these NK cells into Rag2^−/−^γc^−/−^ recipient mice (devoid of NK, T, and B cells) immediately after ICH induction. We found that Poly I:C stimulation induced increased expression of perforin in NK cells and exacerbated ICH injury (Fig. S3). Our results demonstrate that NK cells are a key determinant of early PHE expansion and neurological deterioration after ICH.

### Ablation of NK cell tolerance to cerebral endothelial cells after ICH

**Figure 5.**
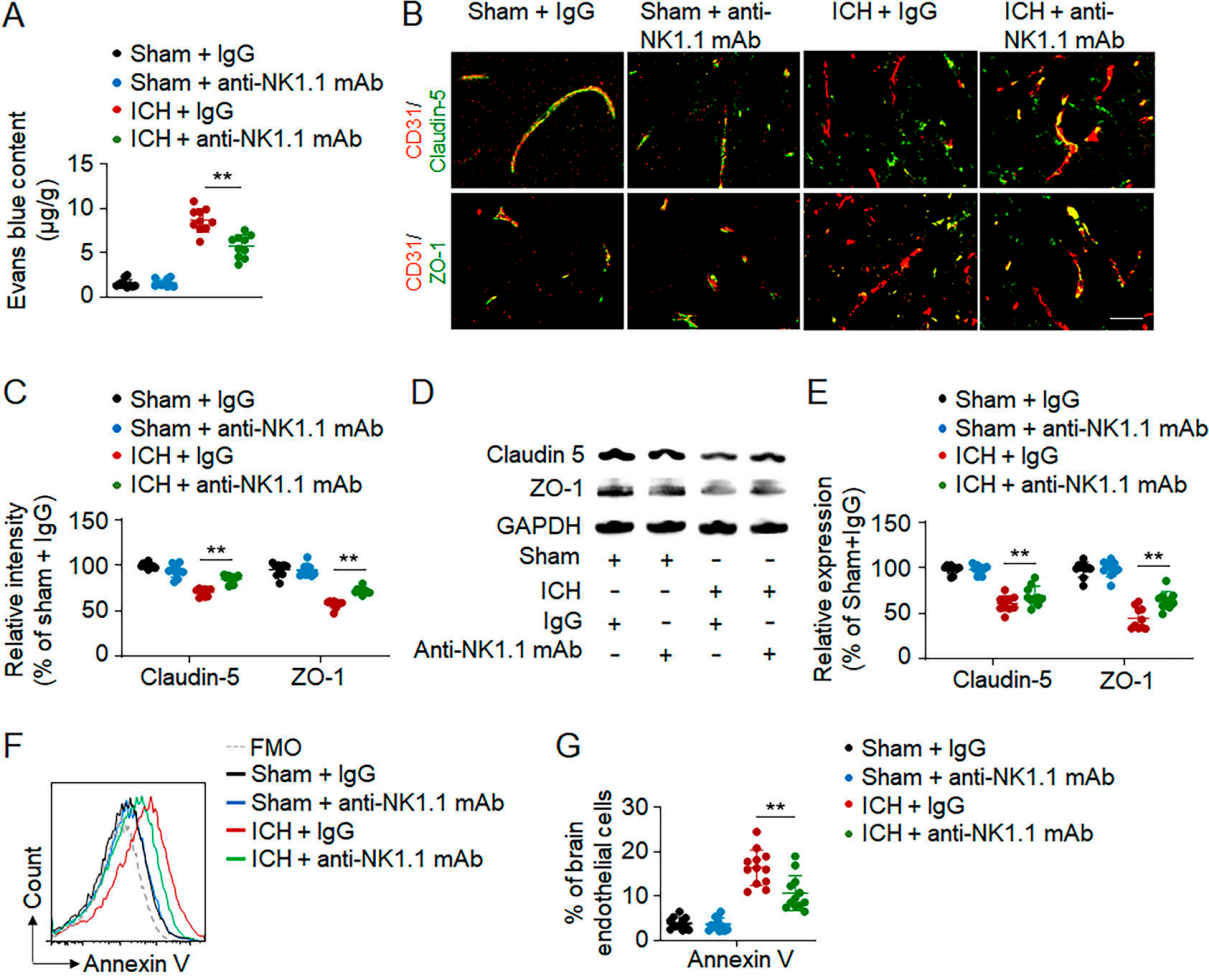
**NK cell depletion preserves BBB integrity and reduces cerebral endothelial cell death after ICH.** ICH was induced by autologous blood injection in C57BL/6 mice. Mouse brain tissues were obtained from groups of sham or ICH mice 1 d after surgery. **(A)** 24 h after ICH, the concentration of Evans blue in mouse brain 3 h after i.v. injection. *n* = 10/group. Data are from three independent experiments. **, P < 0.01. One-way ANOVA. **(B and C)** Images (B) and quantification (C) of the expression of ZO-1 and Claudin 5 in endothelial cells (CD31^+^) in perihematomal brain tissue sections from ICH mice receiving IgG or anti-NK1.1 mAb. Scale bars = 50 µm. *n* = 10/group. Data are from three independent experiments. **, P < 0.01. One-way ANOVA. **(D and E)** Western blot (D) and quantification (E) of ZO-1 and Claudin 5 in the perihematomal tissues after ICH. *n* = 10/group. Data are from three independent experiments. **, P < 0.01. One-way ANOVA. **(F and G)** Flow cytometry analysis (F) and quantification (G) of annexin V expression in brain endothelial cells from ICH mice receiving IgG or anti-NK1.1 mAb. *n* = 12/group. Data are from three independent experiments. **, P < 0.01. One-way ANOVA. Data are presented as mean ± SD.

As cerebral edema is mainly attributed to BBB disruption after ICH (Keep et al., 2012; Qureshi et al., 2009), we determined the impact of NK cells on BBB disruption. Upon depletion of NK cells, we found reduced leakage of Evans blue into the brain parenchyma (Fig. 5 A). Disruption of the BBB involves the breakdown of its tight junctions and endothelial cell damage, both of which were quantified after depletion of NK cells. We found that depletion of NK cells reduced the expression of tight junction proteins (Claudin-5 and ZO-1) in ICH mice (Fig. 5, B–E), accompanied by a decrease in endothelial cell apoptosis (Fig. 5, F and G). In contrast, we found that <5% NK cells expressed annexin V in the ICH brain. These results indicate that NK cells are detrimental to BBB integrity and further damage cerebral endothelial cells after ICH.

**Figure 6.**
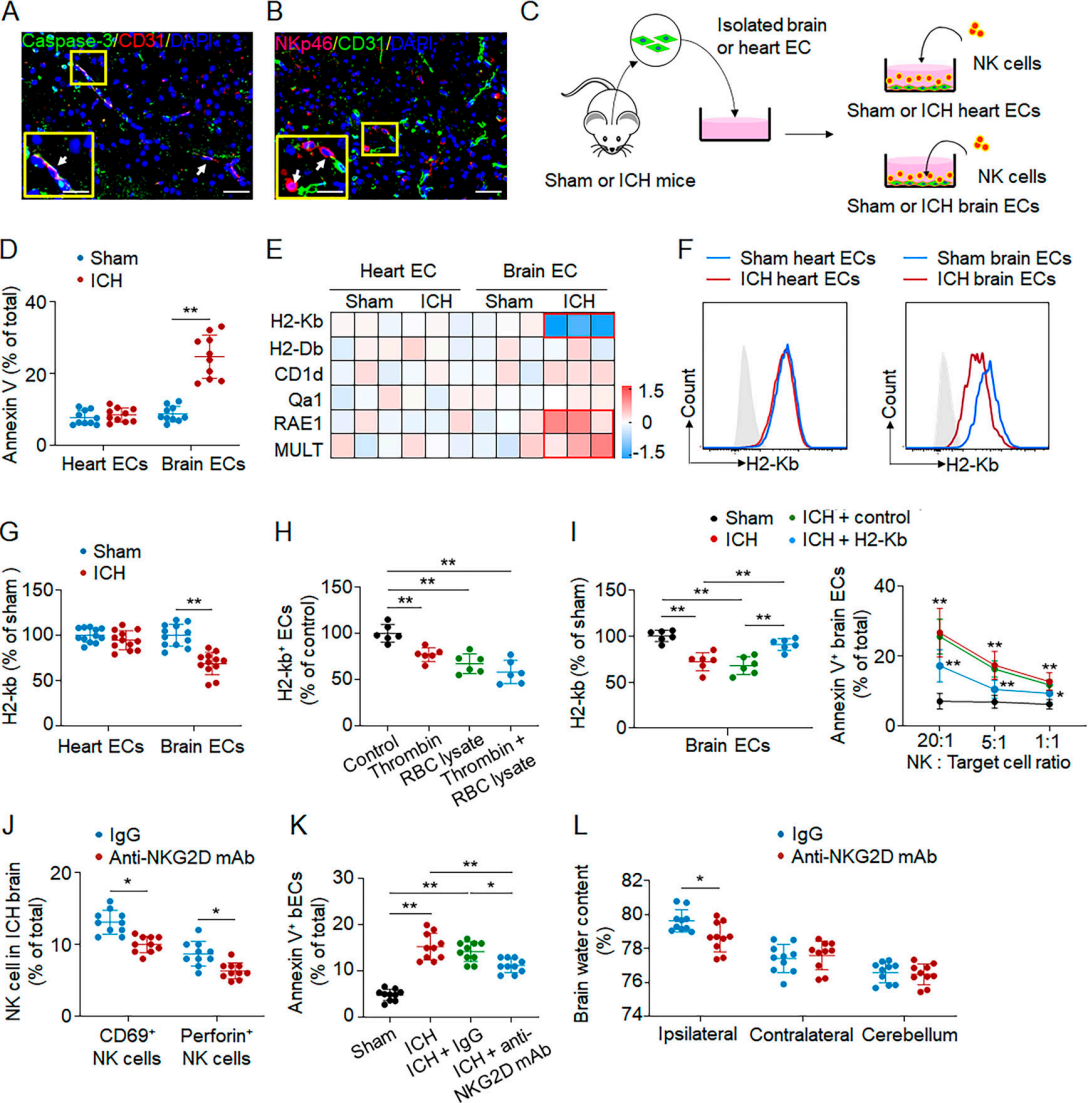
**Ablation of NK cell tolerance toward cerebral endothelial cells after ICH. (A)** Perihematomal brain tissues were obtained from an ICH patient who underwent urgent evacuation of hematoma within 12 h after ictus. Immunostaining of a brain tissue section containing perihematomal regions revealed endothelial cell apoptosis (CD31^+^Caspase-3^+^ cells) in perihematomal regions. Scale bars: 50 µm; 20 µm (inset). **(B)** ICH was induced by autologous blood injection in C57BL/6 mice. Mouse brain tissues were obtained 24 h after ICH. Colocalization of NK cells (NKp46^+^) and endothelial cells (CD31^+^) in perihematomal brain tissues. Scale bars: 50 µm; 20 µm (inset). **(C)** Schematic showing the process for isolating brain endothelial cells (bECs) and heart endothelial cells (hECs) from sham or ICH mice 24 h after surgery. NK cells were from spleen of C57BL/6 mice. **(D)** Flow cytometry analysis of annexin V^+^ cells in indicated groups of endothelial cells. Quantification showing the frequency of annexin V^+^ endothelial cells (CD45^−^annexin V^+^CD31^+^) among total endothelial cells (CD45^−^CD31^+^). *n* = 10/group. Data are from three independent experiments. **, P < 0.01. Unpaired two-tailed *t* test. **(E)** Heatmap showing the expression of MHC-I molecules in endothelial cells from brain and heart tissues 24 h after ICH. MHC-I molecules were measured using flow cytometry. Three independent experiments. Data are shown as the fold change of genes (log2 scale) versus sham group. **(F and G)** Flow cytometry plots and summarized results showing groups of H2-Kb^+^ endothelial cells obtained from brain and heart tissues of ICH mice 24 h after onset. *n* = 12/group. Data are from three independent experiments. **, P < 0.01. Unpaired two-tailed *t* test. **(H)** Brain endothelial cells were isolated from sham mouse brain tissues and then exposed to thrombin and/or RBC lysate for 24 h in vitro. Quantification showing the expression of H2-Kb in groups of brain endothelial cells after exposure to thrombin and/or RBC lysate. *n* = 6/group. Data are from three independent experiments. **, P < 0.01. One-way ANOVA. **(I)** NK cells were harvested from spleen of C57BL/6 wild-type mice. Brain endothelial cells were isolated from ICH mice 24 h after onset. Flow cytometry analysis revealed the expression level of H2Kb on lentivirus-infected cells. An empty lentivirus was used as a control. NK cell killing of brain endothelial cells obtained from sham or ICH mice was measured by cytotoxicity assay. Target cells included groups of bECs from sham mouse brains, bECs from ICH mouse brains, and H2-Kb–overexpressing bECs from ICH mouse brains. *n* = 6/group. Data are from three independent experiments. *, P < 0.05; **, P < 0.01. Left: One-way ANOVA. Right: Two-way ANOVA. **(J–L)** ICH was induced by autologous blood injection. Mice received anti-NKG2D mAb or IgG control immediately after ICH. The counts of brain-infiltrating NK cells expressing CD69 or perforin (J), brain annexin V^+^ endothelial cells (K) and brain edema (L) were measured 1 d after ICH. *n* = 10/group. Data are from three independent experiments. *, P < 0.05; **, P < 0.01. In****K, one-way ANOVA; in L, unpaired two-tailed *t* test. Data are presented as mean ± SD.

Immunostaining results revealed endothelial cell apoptosis in perihematomal regions using brain sections from ICH patients (Fig. 6 A). Because of the acquisition of cytotoxic function in NK cells within the ICH brain and their proximal location to cerebral endothelial cells (Fig. 6 B), we next investigated whether NK cells can directly lyse cerebral endothelial cells. Using an in vitro coculture assay, we found that NK cells induced apoptosis of cerebral endothelial cells that were sorted from ICH but not sham mice (Fig. 6, C and D). In contrast, NK cells did not have a significant impact on cardiac endothelial cells (Fig. 6, C and D). NK cell apoptosis was minimal in this coculture assay. These results suggest that ICH induced a loss of NK cell tolerance to cerebral endothelial cells but not cardiac endothelial cells.

**Figure S4.**
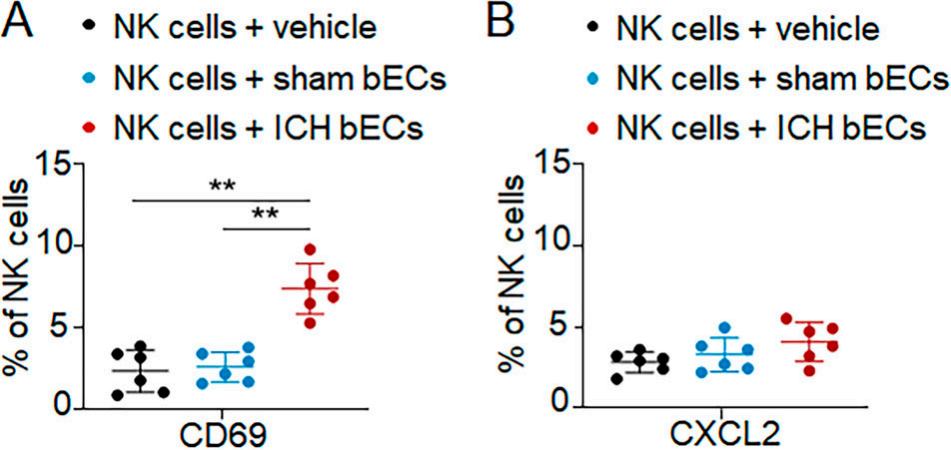
**Effects of brain endothelial cells from ICH brain on NK cells. (A and B)** NK cells were harvested from spleen tissues of C57BL/6 mice. Thereafter, NK cells were cocultured with vehicle, brain endothelial cells (bECs) sorted from ICH C57BL/6 mice, or bECs sorted from sham mice. Flow cytometry analysis showing the expression of CD69 (A) and CXCL2 (B) in NK cells. *n* = 6/group. Data are from three independently repeated experiments. **, P < 0.01. One-way ANOVA. Data are presented as mean ± SD.

The predominant mechanism responsible for NK cell self-tolerance is mediated by inhibitory surface receptors that recognize self MHC-I ligands. We therefore screened the expression of MHC-I molecules in cerebral endothelial cells versus cardiac endothelial cells after ICH. Interestingly, we found that ICH induced a loss of MHC-I ligand H2-Kb, a ligand for the inhibitory NK cell receptor Ly49C, in cerebral endothelial cells derived from ICH mice, accompanied by the up-regulation of MHC-I ligands for NK cell–activating receptors (RAE1 and MULT-1; Fig. 6, E). In contrast, the expression of H2-Kb was unaltered in cardiac endothelial cells sorted from ICH mice (Fig. 6, F and G). Results from in vitro culture of endothelial cells revealed that exposure to RBC lysates and thrombin reduced the expression of MHC I molecule H2Kb (Fig. 6 H). Notably, overexpression of H2-Kb in cerebral endothelial cells sorted from ICH mice reduced NK cell–mediated apoptosis in coculture (Fig. 6 I). In addition, we found that brain endothelial cells obtained from ICH brains can induce up-regulation of CD69 but not CXCL2 in cultured NK cells (Fig. S4, A and B). To assess the in vivo effects of NK cells on endothelial cell death in the ICH brain, we employed an anti-NKG2D mAb to ablate NK cell cytotoxicity response in vivo (Lodoen et al., 2003; Zhu et al., 2010). Injection of anti-NKG2D mAb reduced NK cell activation and cytotoxicity, endothelial cell death, and brain edema in the ICH brain (Fig. 6, J–L). Together, these results suggest that ICH induces ablation of NK cell tolerance to cerebral endothelial cells, which contributes to NK cell–mediated damage of cerebral endothelial cells and BBB disruption.

### Brain-infiltrating NK cells recruit neutrophils and boost focal inflammation after ICH

**Figure 7.**
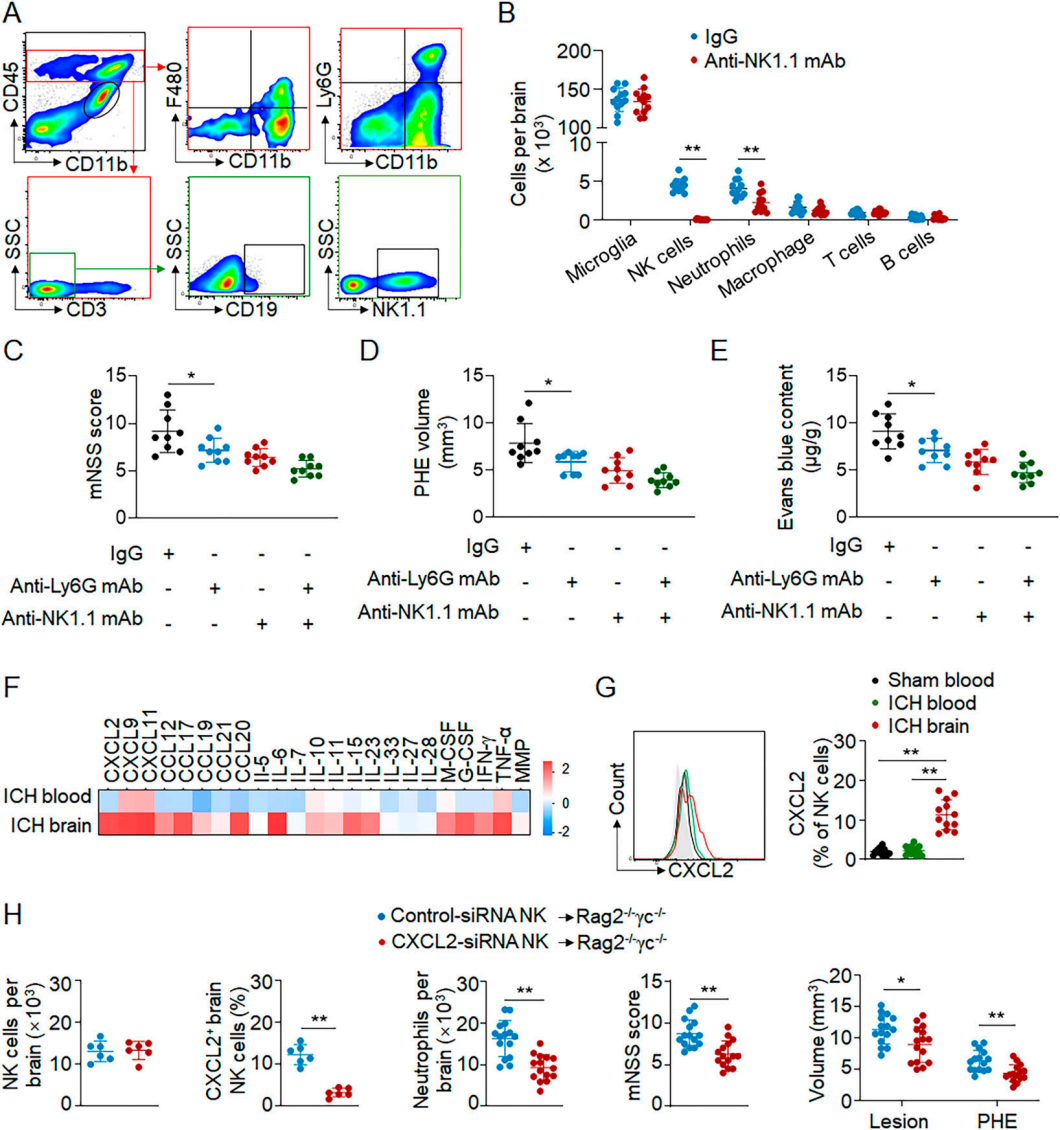
**Brain-infiltrating NK cells recruit neutrophils and augment focal inflammation after ICH. (A–G)** ICH was induced by autologous blood injection in C57BL/6 mice. **(A)** Gating strategy for immune cell populations in the brain, including microglia (CD11b^+^CD45^int^), neutrophils (CD45^high^CD11b^+^Ly6G^+)^, macrophages (CD45^high^CD11b^+^ F4/80^+^), T cells (CD45^high^CD3^+^), B cells (CD45^high^CD3^−^CD19^+^), and NK cells (CD45^high^CD3^−^NK1.1^+^) 12 h after ICH. **(B)** Quantification of indicated immune cell subsets in the brain without hematoma of ICH mice 12 h after ICH. *n* = 12/group. Data are from three independent experiments. **, P < 0.01. Unpaired two-tailed *t* test. **(C–E)** To deplete NK cells, anti-NK1.1 mAb was injected i.p. into each C57BL/6 mouse 24 h before ICH. To deplete neutrophils, 500 µg of anti-Ly6G mAb was injected i.p. into ICH mice immediately after surgery. 24 h after ICH, neurological deficits, PHE volume, and barrier integrity were assessed. Evans blue was used to measure barrier integrity. *n* = 10/group. Data are from three independent experiments. *, P < 0.05. Unpaired two-tailed *t* test. **(F)** Proteome profiler array revealed the top expressed cytokines/chemokines in brain-infiltrating NK cells 12 h after ICH. Data are shown as the fold change of genes (log_2_ scale) versus sham blood group. Three independent experiments. **(G)** Quantification of CXCL2 in NK cells in ICH brain, ICH blood, and sham blood by flow cytometry 12 h after ICH. *n* = 12/group. Data are from three independent experiments. **, P < 0.01. One-way ANOVA. **(H)** ICH was induced by autologous blood injection in Rag2^−/−^γc^−/−^ mice (devoid of T, B, and NK cells). Rag2^−/−^γc^−/−^ mice received adoptive transfer of wild-type NK cells that were treated with control siRNA or CXCL2-siRNA. Quantification showing brain-infiltrating NK cells, CXCL2^+^ brain-infiltrating NK cells, brain-infiltrating neutrophils, mNSS score, lesion volume, and PHE volume in indicated groups of mice 24 h after ICH. *n* = 6 or 15/group. Data are from three independent experiments. *, P < 0.05; **, P < 0.01. Unpaired two-tailed *t* test. Data are presented as mean ± SD.

In addition to cytotoxicity, NK cells also acquired the ability to produce chemokines after homing to the ICH brain. We therefore determined the impact of NK cells on the recruitment of infiltrating immune cell subsets in ICH mice. Flow cytometry analysis revealed that depletion of NK cells led to a significant reduction of brain-infiltrating neutrophils 12 h after ICH (Fig. 7, A and B). In contrast, the counts of microglia (CD11b^+^CD45^int^), T cells (CD45^high^CD3^+^), B cells (CD45^high^CD3^−^CD19^+^), and macrophages (CD45^high^CD11b^+^ F4/80^+^) were not significantly altered (Fig. 7, A and B). These results support the postulation that NK cells facilitate the recruitment of neutrophils into ICH brain.

As major sources of matrix metalloproteinase-9 (MMP-9), ROS, and TNF-α (Wang, 2010), brain-infiltrating neutrophils are predominantly detrimental and exacerbate ICH injury (Moxon-Emre and Schlichter, 2011; Sansing et al., 2011). We also determined the effects of NK cells on neutrophil-induced exacerbation of ICH injury by antibody depletion of neutrophils in ICH mice after NK cell depletion. We found that depletion of neutrophils did not significantly alter neurological deficits, PHE, and barrier disruption in ICH mice subjected to antibody depletion of NK cells (Fig. 7, C–E). These results suggest a critical role of NK cells in cooperation with neutrophils to exacerbate ICH injury.

**Figure S5.**
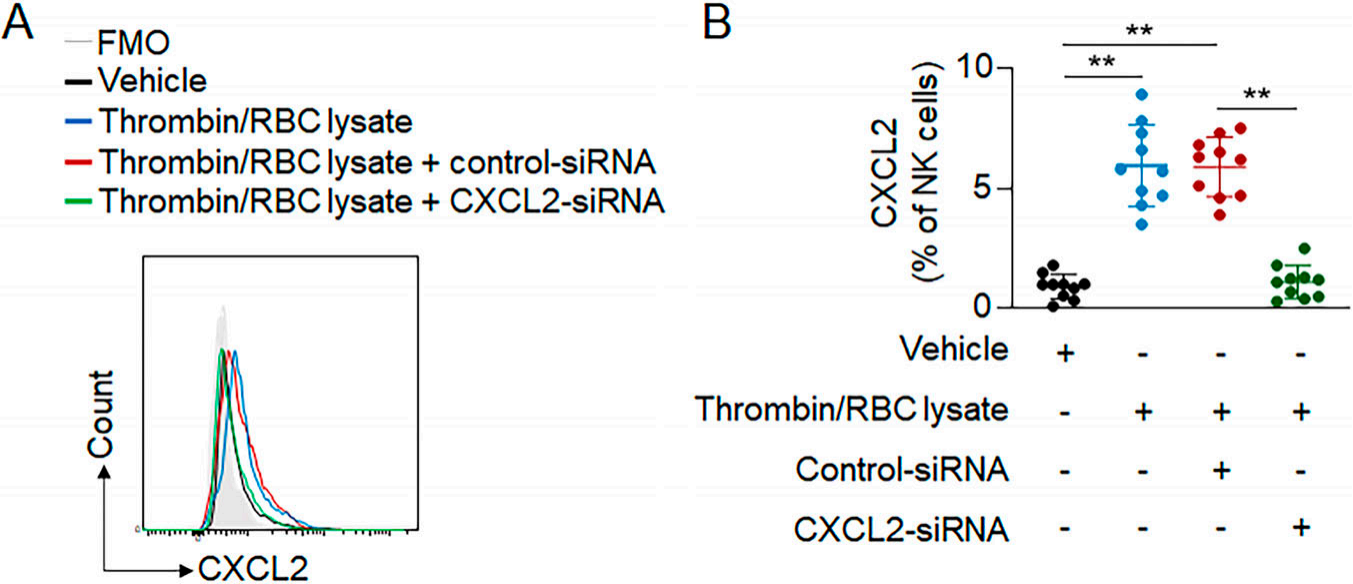
**Expression of CXCL2 in NK cells after exposure to hematoma components. (A)** NK cells were harvested from spleens of C57BL/6 mice and cultured in vitro with control-siRNA or CXCL2-siRNA. Thereafter, NK cells were incubated with thrombin and RBC lysate products. NK cells treated with vehicle were used as control. **(B)** Flow cytometry analysis showing expression of CXCL2 in groups of NK cells receiving indicated treatment. *n* = 10/group. Data are from three independently repeated experiments. **, P < 0.01. Unpaired two-tailed *t* test. Data are presented as mean ± SD.

To investigate the major chemokine responsible for NK cell–mediated recruitment of neutrophils, we assessed the expression profile of immune factors in brain-infiltrating NK cells after ICH. Proteome profiling and flow cytometry analysis revealed that CXCL2, a prominent factor in neutrophil recruitment, was highly up-regulated in brain-infiltrating NK cells (Fig. 7, F and G). Of note, up-regulation of CXCL2 was observed in cultured NK cells after in vitro exposure to thrombin and RBC lysate (Fig. S5, A and B), suggesting that exposure to the breakdown products of hematoma is sufficient to up-regulate CXCL2 in NK cells.

We next investigated the impact of NK cell–derived CXCL2 on the recruitment of neutrophils in ICH. To this end, we performed genetic knockdown of CXCL2 in cultured NK cells using siRNA (Fig. S5, A and B), and then transferred these NK cells to Rag2^−/−^γc^−/−^ mice (devoid of T, B, and NK cells) immediately after ICH. We found that the number of brain-infiltrating neutrophils was significantly reduced in mice receiving NK cells treated with CXCL2 siRNA versus control siRNA (Fig. 7 H), accompanied by a decrease of neurological deficits, lesion volume, and PHE volume (Fig. 7 H). Together, these findings suggest that NK cells recruit neutrophils into ICH brain, leading to accelerated PHE expansion and neurological deficits.

## Discussion

The initiation and formation of PHE lead to rapid neurological deterioration starting from minutes to hours after ICH. Considering that the majority of PHE volume occurs within 24 h after onset and the temporal profiles of the inflammatory responses, only immune cells that can be activated without the requirement of antigen priming could respond rapidly to acute ICH and contribute to early PHE formation. Unlike adaptive immune cells that need classic antigen presentation for activation, NK cells possess these characteristics. Indeed, here we identify swift arrival of NK cells to the hemorrhagic brain before other mobilized immune cells, including neutrophils and T cells. Single-cell RNA-seq and unbiased clustering analysis show functional alternations of chemokine production and cytotoxicity in these brain-infiltrating NK cells. These results suggest that the ICH brain renders NK cells with cytotoxic and proinflammatory features, contributing to early PHE formation and brain edema.

In addition to the direct cytotoxicity to BBB following ICH, we further report that NK cells cooperate with neutrophils to damage the BBB. This finding demonstrates the synergistic capacity of brain-infiltrating NK cells in producing immune factors and orchestration of focal inflammation with other infiltrating immune cells. Unlike a previous study in murine models of ICH demonstrating the counts of lymphocyte subsets in the whole-brain tissues at ≥24 h after onset (Mracsko et al., 2014), our results revealed early arrival of NK cells in the perihematoma regions, within 12 h of ICH onset. Notably, we found NK cells expressing CD69 or perforin in the ICH brain. These findings support the pivotal role of NK cells in focal inflammation and PHE expansion. As blood draws from ICH patients were performed at later time points versus the early time points for collection of brain tissues during emergent hematoma evacuation in this study, future studies should evaluate NK cells in the brain versus blood at similar time points after ICH. Besides the recruited immune cells, the neurovascular unit also constitutes resident immune cells, including glia and perivascular macrophages. Whether and how NK cells interact with these brain-intrinsic cell types to impact PHE formation is of interest and warrants future investigation. Nevertheless, the results presented in this study illustrate how NK cells orchestrate focal inflammation and contribute to the genesis of PHE. In line with these findings, previous studies report that NK cells can promote focal inflammation and neural injury in the setting of ischemic stroke and traumatic brain injury (Gan et al., 2014; Kong et al., 2014; Li et al., 2017b; Zhang et al., 2014). Although the cellular and biochemical cascades that activate the immune system differ among brain ischemia, traumatic brain injury, and ICH pathologies, the early detrimental role of NK cells in these disease types suggest that cell-mediated immunity might be a promising target to attenuate acute brain insults. Of interest, NK cell depletion leads to reduced recruitment of neutrophils more than other immune cell types, although brain-infiltrating NK cells also express CXCL9 and CXCL11, which may contribute to the recruitment of other immune cell types. This finding suggests that NK cells may not be the only cellular players that can recruit immune cell subsets other than neutrophils. It is also possible that NK cells may not be the only source of CXCL9 and CXCL11 in the brain. Future studies are required to reveal the source and types of chemokines that underpin the recruitment of specific immune cell subsets after ICH.

After ICH, we observed transcriptional changes in NK cells after homing to the brain. These results highlight that the fate and function of NK cells are determined by focal environmental factors. Within the ICH brain, infiltrating NK cells exhibit increased cytotoxicity and chemokine production versus peripheral NK cells. Upon entering the hemorrhagic brain, NK cells are exposed RBC lysate products and thrombin, which leach from the hematoma, as well as subsequent danger signals excreted from injured brain tissue. The adaptation to the ICH brain grants NK cells cytotoxic and proinflammatory properties that accelerate PHE formation. The finding of endothelial cell–induced NK cell activation suggests the cross-talk between injured endothelial cells and NK cells that contributes to amplified endothelial cell damage and barrier disruption in the ICH brain. Supplementing this interpretation, gene enrichment analysis of brain-infiltrating NK cells revealed an array of immune and metabolic pathways that are activated by environmental factors, including key immune factors such as CXCL2, CCL2, TNF, and IL-1, that are reported to be dramatically elevated in the ICH brain (Fu et al., 2015; Keep et al., 2012; Mracsko and Veltkamp, 2014). Other than NK cells, single-cell mRNA profiling of other brain-infiltrating lymphocyte subsets including cytotoxic T cells would also be interesting, and awaits further investigation in future studies. Nevertheless, the altered gene expression of brain-infiltrating NK cells supports that environmental factors within the ICH brain shape NK cell response. Additionally, it remains unclear whether the altered transcriptional profiles in brain NK cells result from a unique environment in the brain or a similar inflammatory environment that could also occur in other organs after injury. Future studies regarding this aspect are critical to reveal brain-specific features of NK cells after neural injury.

Currently, medical management of most ICH patients includes supportive measures using nonspecific osmotherapeutic drugs (mannitol, glycerin fructose, albumin, etc.) or hypertonic saline infusion, which have not been demonstrated as effective therapies to improve outcomes in ICH patients (Aronowski and Zhao, 2011; Urday et al., 2015; Zheng et al., 2016). The benefits of surgical hematoma evacuation and pharmacologic ion chelation using deferoxamine remain uncertain (Hanley et al., 2019; Selim et al., 2019). Recently, we and others reported the efficacy of immune modulator fingolimod, which reduces PHE expansion and improves neurological outcome after ICH in preclinical and proof-of-concept clinical studies (Fu et al., 2014, 2015). Fingolimod is a sphingosine 1-phosphate receptor (S1PR) modulator that inhibits the egress of lymphocytes (in which NK cells are a major subtype) from lymph nodes, prevents their recirculation, and reduces the trafficking of lymphocytes into the brain (Aktas et al., 2010; Brinkmann et al., 2010). Considering the potent cytotoxic and proinflammatory capacity of NK cells, these results suggest that prevention of NK cell infiltration into the brain might be a viable approach to reduce brain inflammation and attenuate PHE formation after ICH. In light of these studies, a randomized, placebo-controlled, subject- and investigator-blinded trial of another S1PR modulator, siponimod, in ICH patients is ongoing (NCT03338998; Bobinger et al., 2019). Although the benefits of S1PR modulation in improving outcome following ICH awaits more clinical evidence, immune modulation targeting lymphocytes to prevent PHE formation and neurological deterioration deserves further investigation.

## Materials and methods

### Human brain tissues

Brain tissues were acquired from patients with ICH who required surgery to evacuate the hematoma or patients who died from nonneurological diseases (controls). Informed consent was obtained from each patient or a legally defined surrogate. Collection of human brain tissue was performed according to protocols approved by the institutional review board of Tianjin Medical University General Hospital (Tianjin, China) or the Third People's Hospital of Datong (Shanxi, China). Among 18 cases studied, 9 were from patients with ICH (Table S1). These patients had primary ICH with symptom onset <12 h before surgery (seven male; two female). Several inclusion and exclusion criteria were designated. Enrolled ICH patients were aged ≥18 yr with hemorrhage volume of 50–100 ml measured by computed tomography. Exclusion criteria were autoimmune diseases, preexisting brain diseases, and infections, and concomitant use of immunosuppressive or immune-modulating therapies. The nine control tissues were from patients who died from nonneurological diseases (six male; three female). Histopathologic examination confirmed no pathological changes in brain sections beyond those expected in control subjects with nonneurological diseases. Patients with ICH and controls did not differ significantly in terms of mean age at death (ICH patients, 58 ± 4.1; control, 54.9 ± 4.5; mean ± SEM; P > 0.05, Student’s *t* test).

### Human blood samples

Nine patients with ICH and nine age-matched control subjects were enrolled (Table S1). Blood samples were collected 24 h after ICH onset (seven male, two female). Blood samples of healthy individuals were used as controls (six male, three female). There was no significant age difference between ICH patients and control subjects (ICH patients, 58.4 ± 4.1; control, 59.4 ± 3.2; mean ± SEM; P > 0.05, Student’s *t* test). Enrolled ICH patients were aged ≥18 yr with hemorrhage volume of 50–100 ml measured by computed tomography. Exclusion criteria include medical history of autoimmune diseases, preexisting brain diseases and infection, and concomitant use of immunosuppressive or immune-modulating therapies. Control subjects were chosen on the basis of standardized inclusion and exclusion criteria as follows. Inclusion criteria were (1) age ≥18; (2) normal basic laboratory tests; (3) and normal neurological function on neurological examination. Exclusion criteria were (1) neurologic or psychiatric disorder; (2) history of tumor; (3) history of drug or alcohol abuse; and (4) history of medication use including central nervous system stimulants, antiepileptic drugs, cortisone, and insulin. The study protocol and supporting documents were approved by the institutional review board of Tianjin Medical University General Hospital (Tianjin, China) or the Third People's Hospital of Datong (Shanxi, China). Written informed consent was obtained from each patient or legal surrogate.

### Mice

10–12-wk-old male mice were used in this study. C57BL/6 mice were purchased from Charles River Laboratories. Rag2^−/−^γc^−/−^ mice were purchased from Taconic. CD1d^−/−^ mice were purchased from the Jackson Laboratory. All mutant mice were backcrossed to the C57BL/6 background for 8–12 generations. Mice were housed no more than five animals per cage in pathogen-free conditions under a standardized light–dark cycle with free access to food and water. For all experiments, age-matched male littermates were used between experimental groups. Animal surgeries were performed under anesthesia. All animal experiments were approved by the Committee on the Ethics of Animal Experiments of Tianjin Neurological Institute (Tianjin, China). All experiments were conducted in accordance with the National Institutes of Health Guidelines for the Care and Use of Laboratory Animals and were designed and performed according to the Animal Research: Reporting In Vivo Experiments guidelines (https://arriveguidelines.org/).

### ICH models

ICH was induced in mice by injection of autologous blood or collagenase as previously described (Li et al., 2017a). Mice were anesthetized using 1–3% isoflurane inhalation and fixed on a stereotactic frame. A burr hole was drilled on the right side of skull at 2.3 mm lateral to midline and 0.5 mm anterior to bregma. For the autologous blood model, 30 µl of nonheparinized autologous blood was withdrawn from the angular vein. The first 5 µl blood was injected at a rate of 1 µl/min at a depth of 3 mm beneath the hole. Thereafter, the needle was moved to a depth of 3.7 mm and paused for 5 min. The remaining 25 µl of blood was injected at the same rate of 1 µl/min. For the collagenase model, 0.0375 U bacterial collagenase was dissolved in 0.5 µl of saline and infused to the striatum (0.5 mm anterior, 2.3 mm left lateral, and 3.5 mm deep relative to bregma) at the speed of 0.5 µl/min. Sham controls were injected with an equal volume of saline. Throughout the procedure, animal body temperature was maintained at 37°C with a homeothermic blanket. After surgery, animals remained under observation with free access to food and water.

### In vivo antibody administration

Anti-NK1.1 (PK136) mAb was purchased from BioLegend. Anti-Ly6G (1A8) mAb was purchased from Bio X Cell. Mouse IgG2a (Sigma-Aldrich) was used as the isotype control antibody. For in vivo depletion of NK1.1^+^ cells, 200 µg of anti-NK1.1 mAb was injected i.p. into each mouse. Depletion of NK1.1^+^ cells was confirmed by flow cytometry and consistently achieved >95% depletion. For in vivo depletion of neutrophils, 500 µg of anti-Ly6G mAb was injected i.p. into each mouse immediately after ICH.

### Neurological function assessment

Neurological function assessment was performed by two investigators who were blinded to the treatment groups. The modified Neurological Severity Score (mNSS), corner turn test, and rotarod test were conducted to evaluate neurological deficits of ICH mice at defined time points, as previously described (Li et al., 2017a, 2017c).

#### mNSS

In the neurological deficit scoring system, mice were evaluated for motor function (muscle and abnormal movement), sensory function (visual, tactile, and proprioceptive), and reflexes (pinna, corneal, and startle). The range of scores is from 0 to 18, defined as follows: a score of 13–18 indicates severe injury, 7–12 indicates moderate injury, and 1–6 indicates mild injury.

#### Corner-turning test

The corner-turning test was conducted to evaluate sensorimotor and postural asymmetries. Mice were allowed to proceed into a corner with an angle of 30° and then had to turn right or left. Each mouse repeated this procedure 10 times with an interval of ≥30 s between trials. The percentage of ipsilateral turns was then calculated.

#### Rotarod test

The rotarod test was conducted to evaluate motor coordination and balance. Mice were trained for 1 wk before ICH induction. At indicated time points after ICH, mice were placed on a rotarod apparatus. The rotating rod was 3-cm diameter with a nonslippery surface, 30 cm in length, and placed at height of 20 cm from the base. Each mouse was placed on the rod at a speed of 4 rotations per minute (rpm) which accelerated over the course of 300 s to 40 rpm. The duration of each mouse on the rod was recorded automatically. Each mouse was tested in three consecutive trials with an interval of 15 min. The results were calculated as the average of three trials.

### Neuroimaging

7T small-animal magnetic resonance imaging (MRI) scans (Bruker Corp.) were used as previously described (Gan et al., 2014; Liu et al., 2017). T2-weighted imaging was performed to assess total lesion volume. The setup parameters were as follows: repetition time, 4,500 ms; echo time, 65.5 ms; field of view, 28 × 28 mm^2^; image matrix, 256 × 256; slice thickness, 0.5 mm. Susceptibility weighted imaging was used to measure hematomas. The setup parameters were as follows: repetition time, 30 ms; echo time, 10 ms; flip angle, 25°; field of view, 32 × 32 × 16 mm^3^; image matrix, 256 × 256. The volumes were manually outlined and calculated by multiplying the sum of the volume by the distance between sections (0.5 mm) using MIPAV software. PHE volumes were calculated as total lesion volume minus hematoma volume. MRI data were analyzed by two investigators blinded to experimental groups.

### Brain water content assessment

Brain water content was measured on day 1 after ICH, as previously described (Li et al., 2017a, 2017c; Ren et al., 2018). Briefly, without perfusion, brain tissue was removed and divided into three parts: ipsilateral hemisphere, contralateral hemisphere, and cerebellum. Brain tissues were weighed to measure wet weights and then dried for 24 h at 100°C to obtain dry weights. The following formula was used to calculate brain water content: (wet weight − dry weight)/wet weight × 100%.

### Flow cytometry

Single-cell suspensions were prepared and stained with fluorochrome-conjugated antibodies as previously described (Li et al., 2017b; Liu et al., 2016, 2017). All antibodies were purchased from BioLegend or BD Bioscience, unless stated otherwise. Antibodies were directly labeled with one of the following fluorescent tags: FITC, PE, PerCP-Cy5.5, or allophycocyanin. The following antibodies were used: anti-human CD3 (HIT3A), CD56 (MEM-188), CD69 (FN50), CD49a (SR84), CD57 (NK-1), and perforin (B-D48, Abcam); anti-mouse CD3 (17A2), NK1.1 (PK136), perforin (S16009A), CD69 (H1.2F3), Annexin V, H2-Kb (AF6-88.5), H2-Db(KH95), CD1d (1B1), Qa1 (6A8.6F10, Santa Cruz), RAE-1 (CX1), MULT1 (5D10, eBioscience), CD11b (M1/70), CD45 (30-F11), F4/80 (BM8), CD19 (1D3/CD19), and Ly6G (1A8). CXCL2 was quantified using an unconjugated CXCL2 antibody (R&D Systems) and an Alexa Fluor 488–conjugated antibody (Invitrogen). Flow cytometric measurements were performed on a FACS Aria III (BD Bioscience) and analyzed using Flowjo v7.6 software (Informer Technologies).

### Single-cell RNA-seq of NK cells

Single-cell suspensions were prepared from pooled mouse blood and brain tissues 12 h after ICH induced by autologous blood injection or sham operations. CD3^−^NK1.1^+^NKp46^+^ cells were isolated at 4°C using FACS, and cell viability was ≥90% for all samples by CountStar Rigel. Single-cell suspensions of ∼12,000 cells were captured from each sample on an array of >200,000 microwells. Beads with oligonucleotide barcodes were added to saturation so that cells could be paired with beads in microwells. Captured cells were lysed, and released RNA was barcoded through reverse transcription in individual microwells. Reverse transcription was performed on a ThermoMixer C (Eppendorf) at 1,200 rpm and 37°C for 45 min. cDNA was generated, amplified using 22 cycles of PCR, and quality assessed using an Agilent 4200.

Single-cell RNA-seq libraries were constructed using BD Rhapsody WTA Amplification Kit. The libraries were finally sequenced using an Illumina Novaseq6000 sequencer with a sequencing depth of ≥100,000 reads per cell with pair-end 150-bp reading strategy. The BD Rhapsody analysis pipeline was used to process sequencing data (.fastq files). Cell labels and molecular indices were identified, and gene identity was determined by alignment against the GRCm38 Genome Reference. A table containing molecule counts per gene per cell was the output. Gene expression profiles of 12,443, 8,751, and 4,270 NK cells were recovered for sham blood, ICH blood, and ICH brain samples. Analysis of the single-cell transcriptome profiles was performed using BD Data View (BD Biosciences). The GEO accession number for the RNA-seq data is GSE155275.

### Immunostaining

Brian tissue sections were incubated overnight with primary antibodies at 4°C and then with corresponding fluorochrome-conjugated secondary antibodies at room temperature for 1 h. The following primary antibodies were used: anti-human NKp46 (195314; R&D Systems), CD49a (SR84; BD Bioscience), CD57 (MA1-81071; Invitrogen), CD68 (ED1; Abcam), CD4 (H-370; Santa Cruz), CD8 (UCH-T4; Santa Cruz), CD19 (HIB19; BioLegend), CD16b (CLB-gran11.5; BD Bioscience), CD66b (polyclonal; Bioss), perforin (dG9; BioLegend), CD69 (D-3; Santa Cruz), Caspase-3 (9661; CST), CD31 (JC/70A; Abcam); anti-mouse NKp46 (M20; Santa Cruz), CD49a (Ha31/8; BD Bioscience), ly6G (1A8; BioLegend), TMEM119 (28-3; Abcam), CD68 (ED1; Abcam), CD8 (53-6.7; eBioscience), CD4 (GK1.5; eBioscience), CD19 (1D3; BD Bioscience), CD31 (polyclonal; Abcam), claudin5 (4C3C2; Invitrogen), and ZO-1 (ZO-1-1A12; Invitrogen). The following fluorochrome-conjugated secondary antibodies were used: donkey anti-rabbit 488 (1:1,000; Invitrogen), donkey anti-rabbit 546 (1:1,000; Invitrogen), donkey anti-goat 546 (1:1,000; Invitrogen), donkey anti-mouse 594 (1:1,000; Invitrogen), donkey anti-mouse 488 (1:1,000; Invitrogen), goat anti-rat 488 (1:1,000; Invitrogen), and goat anti-rat 555 (1:1,000; Invitrogen). Images were acquired on a fluorescence microscope (Olympus BX-61).

### Cell isolation, RNA interference, and passive transfer of NK cells

NK cells were sorted from pooled splenocytes of naive C57BL/6 mice (BD Bioscience). NK cells were purified using magnetic beads (Miltenyi Biotech) coupled with two rounds of cell sorting on a FACS Aria III system. The purity of NK cells was confirmed with flow cytometry after sorting (>98%). NK cells were cultured in RPMI medium with 10% FBS, 1% penicillin/streptomycin, 10 µg/ml IL-15, and 10 µg/ml IL-2.

siRNA was used to target *Cxcl2* (Santa Cruz). The siRNA components consist of three target-specific 19–25-nt siRNAs designed to target *Cxcl2*. A scrambled siRNA plasmid was used as a control (Santa Cruz). NK cells were transfected with 60 pmol of *Cxcl2*–siRNA or control fragments mixed with 2 µl of Lipofectamine 2000 (Invitrogen) according to the manufacturer’s instructions. After transfection, NK cells were cultured for an additional 24 h before use. 2 × 10^6^ NK cells were injected via the tail vein into Rag2^−/−^γc^−/−^ recipient mice immediately after ICH.

### Endothelial cell culture, NK cell cytotoxicity assay, and H2-Kb overexpression with lentivirus

Endothelial cells were isolated from mouse heart and brain tissues. Mouse brain and heart tissues were collected 24 h after ICH induced by autologous blood injection or sham operations. Tissues were then minced with scissors in ice-cold DMEM (Invitrogen). Subsequently, the minced tissues were digested with 1 mg/ml collagenase I (Sigma-Aldrich) at 37°C for 30 min in tubes in a water bath. After enzyme digestion, the reaction was stopped by adding inactivated FBS to the medium. After filtering and centrifugation, cells were resuspended in 1% BSA buffer and stained with anti-CD31 and anti-CD45 antibodies at 4°C for 30 min. Thereafter, endothelial cells (CD45^−^CD31^+^ cells) were sorted using a FACS Aria III flow cytometer (BD Bioscience). The purity of sorted endothelial cells was >98%. NK cells were isolated from spleen tissues of wild-type mice and then cultured in medium containing 10 µg/ml IL-15 and 10 µg/ml IL-2. Sorted endothelial cells were cocultured with NK cells at effector:target ratios of 20:1, 5:1, and 1:1 for 5 h. The apoptosis of endothelial cells (CD45^−^CD31^+^) was measured using annexin V (BioLegend).

H2-Kb expression with lentivirus in cultured endothelial cells was performed as previously described, with modification (Gan et al., 2014; Liu et al., 2017). Full-length murine H2-Kb was amplified by RT-PCR from RNA of murine endothelial cells. After verification of the sequence, cDNA was inserted in the lentivirus vector under the promotor of CMV. The recombinant lentivirus was produced by Lenti-X 293T cells (Takara Bio) and collected 72 h after transfection. The titers were up to 5–8 × 10^8^ infectious U/ml. Cultured endothelial cells were infected with virus in 12-well plates. Thereafter, the virus-containing transduction medium was replaced with fresh growth medium. Endothelial cell cultures were incubated for 48 h at 37°C and 5% CO_2_ before NK cell cytotoxicity assay.

For thrombin and RBC lysate treatment, cultured NK cells or endothelial cells were exposed to RBC-lysate (1 µl lysate/100 µl medium) and thrombin (3 U/ml) for 24 h. Thereafter, cultured cells were harvested and followed by flow cytometry analysis. The thrombin was LPS-free and confirmed using an LPS ELISA kit (LSBio).

### Proteome profiler mouse cytokine array

NK cells were purified from mouse blood and brain tissues 12 h after ICH or sham operations. Thereafter, NK cells were lysed with cell lysis buffer (R&D Systems) supplemented with protease inhibitor mixture (Sigma-Aldrich) at 4°C for 30 min. Total protein was quantified using a protein bicinchoninic acid kit (Thermo Fisher Scientific). A Mouse XL Cytokine Array Kit (R&D Systems) was used to measure cytokine and chemokine levels. Immunospots were captured using a Gel Doc Imager (Bio-Rad), and spot density was measured using ImageJ software (National Institutes of Health).

### Western blot

On day 1 after ICH induced by autologous blood injection, perihematomal tissues were isolated from mouse brains. Total proteins were extracted using radioimmunoprecipitation assay lysis buffer (Solarbio) supplemented with protease inhibitor mixture tablets (Roche). Total protein was determined using a bicinchoninic acid Protein Assay Kit (Solarbio). Equal amounts of protein were separated by SDS-PAGE and transferred to PVDF membranes (Merck). Membranes were blocked with 5% nonfat milk in Tris-buffered saline containing 0.1% Tween-20 and probed with primary antibodies against anti-ZO-1 (1:1,000; Invitrogen); anti-claudin-5 (1:1,000; Invitrogen), and anti-GADPH (1:1,000; Cell Signaling Technology) at 4°C overnight. The membranes were washed and incubated with appropriate horseradish peroxidase–linked secondary antibodies (1:5,000; Transgen Biotech) for 1 h. The relative intensity of protein signals was normalized to corresponding loading controls and quantified by densitometric analysis with ImageJ.

### Statistics

Data are presented as mean ± SD. The exact values of sample size (*n*) are given in figure legends and represent either the number of animals used in vivo or the number of cell cultures used in vitro. The sample size was determined by power analysis using α = 0.05 with 80% power to detect statistical differences. Power calculations for experiments were conducted using SAS 9.1 software (SAS Institute). The experimental design was based on previous publications with similar mechanistic studies done in our laboratory (Li et al., 2017b; Liu et al., 2016, 2017). All animals in experimental and control groups were littermates. Experimental groups, data collection, and data analysis were blinded by using different investigators or masking sample labels. All experiments with animals and cell cultures were randomly assigned to experimental groups. Sample exclusion was done as a result of mouse death after surgery. Experiments of single-cell RNA-seq were successfully reproduced at least twice. All other experiments presented were successfully reproduced at least three times. For each set of data compared, we evaluated normality using the Kolmogorov–Smirnov test. Two-tailed unpaired Student’s *t* test was used to determine significant differences between two groups. One-way ANOVA followed by Tukey’s post hoc test was used for comparisons of three or more groups. Two-way ANOVA followed by Bonferroni’s posttests was used for multiple comparisons. A statistical false discovery rate value of <0.05 was used for all RNA-seq analyses. Values of P < 0.05 were considered significant. Statistical analyses were performed using Prism 7.0 software (GraphPad).

### Online supplemental material

Fig. S1 shows enrichment of immune signatures in brain-infiltrating NK cells after ICH. Fig. S2 shows in vivo NK cell depletion using an anti-NK1.1 mAb. Fig. S3 shows effects of Poly I:C stimulation of NK cells on ICH injury. Fig. S4 displays the effects of brain endothelial cells from ICH brain on NK cells. Fig. S5 exhibits CXCL2 expression in NK cells after exposure to hematoma components. Table S1 lists patient characteristics.

## Supplementary Material

Table S1 shows patient characteristics.

## References

[bib1] Aktas O., Küry P., Kieseier B., and Hartung H.P. 2010 Fingolimod is a potential novel therapy for multiple sclerosis. Nat. Rev. Neurol. 6:373–382. 10.1038/nrneurol.2010.76 20551946

[bib2] Aronowski J., and Zhao X. 2011 Molecular pathophysiology of cerebral hemorrhage: secondary brain injury. Stroke. 42:1781–1786. 10.1161/STROKEAHA.110.596718 21527759 PMC3123894

[bib3] Bobinger T., Manaenko A., Burkardt P., Beuscher V., Sprügel M.I., Roeder S.S., Bäuerle T., Seyler L., Nagel A.M., Linker R.A., 2019 Siponimod (BAF-312) Attenuates Perihemorrhagic Edema And Improves Survival in Experimental Intracerebral Hemorrhage. Stroke. 50:3246–3254. 10.1161/STROKEAHA.119.027134 31558140

[bib4] Brinkmann V., Billich A., Baumruker T., Heining P., Schmouder R., Francis G., Aradhye S., and Burtin P. 2010 Fingolimod (FTY720): discovery and development of an oral drug to treat multiple sclerosis. Nat. Rev. Drug Discov. 9:883–897. 10.1038/nrd3248 21031003

[bib5] Crinier A., Milpied P., Escalière B., Piperoglou C., Galluso J., Balsamo A., Spinelli L., Cervera-Marzal I., Ebbo M., Girard-Madoux M., 2018 High-Dimensional Single-Cell Analysis Identifies Organ-Specific Signatures and Conserved NK Cell Subsets in Humans and Mice. Immunity. 49:971–986.e5. 10.1016/j.immuni.2018.09.009 30413361 PMC6269138

[bib6] Fu Y., Hao J., Zhang N., Ren L., Sun N., Li Y.J., Yan Y., Huang D., Yu C., and Shi F.D. 2014 Fingolimod for the treatment of intracerebral hemorrhage: a 2-arm proof-of-concept study. JAMA Neurol. 71:1092–1101. 10.1001/jamaneurol.2014.1065 25003359

[bib7] Fu Y., Liu Q., Anrather J., and Shi F.D. 2015 Immune interventions in stroke. Nat. Rev. Neurol. 11:524–535. 10.1038/nrneurol.2015.144 26303850 PMC4851339

[bib8] Gan Y., Liu Q., Wu W., Yin J.X., Bai X.F., Shen R., Wang Y., Chen J., La Cava A., Poursine-Laurent J., 2014 Ischemic neurons recruit natural killer cells that accelerate brain infarction. Proc. Natl. Acad. Sci. USA. 111:2704–2709. 10.1073/pnas.1315943111 24550298 PMC3932858

[bib9] Hanley D.F., Thompson R.E., Rosenblum M., Yenokyan G., Lane K., McBee N., Mayo S.W., Bistran-Hall A.J., Gandhi D., Mould W.A., ; MISTIE III Investigators 2019 Efficacy and safety of minimally invasive surgery with thrombolysis in intracerebral haemorrhage evacuation (MISTIE III): a randomised, controlled, open-label, blinded endpoint phase 3 trial. Lancet. 393:1021–1032. 10.1016/S0140-6736(19)30195-3 30739747 PMC6894906

[bib10] Iadecola C., and Anrather J. 2011 The immunology of stroke: from mechanisms to translation. Nat. Med. 17:796–808. 10.1038/nm.2399 21738161 PMC3137275

[bib11] Kaur G., Trowsdale J., and Fugger L. 2013 Natural killer cells and their receptors in multiple sclerosis. Brain. 136:2657–2676. 10.1093/brain/aws159 22734127 PMC3754456

[bib12] Keep R.F., Hua Y., and Xi G. 2012 Intracerebral haemorrhage: mechanisms of injury and therapeutic targets. Lancet Neurol. 11:720–731. 10.1016/S1474-4422(12)70104-7 22698888 PMC3884550

[bib13] Kong X.D., Bai S., Chen X., Wei H.J., Jin W.N., Li M.S., Yan Y., and Shi F.D. 2014 Alterations of natural killer cells in traumatic brain injury. Neurosci. Bull. 30:903–912. 10.1007/s12264-014-1481-9 25446874 PMC5562566

[bib14] Lee K.R., Kawai N., Kim S., Sagher O., and Hoff J.T. 1997 Mechanisms of edema formation after intracerebral hemorrhage: effects of thrombin on cerebral blood flow, blood-brain barrier permeability, and cell survival in a rat model. J. Neurosurg. 86:272–278. 10.3171/jns.1997.86.2.0272 9010429

[bib15] Li M., Li Z., Ren H., Jin W.N., Wood K., Liu Q., Sheth K.N., and Shi F.D. 2017 a Colony stimulating factor 1 receptor inhibition eliminates microglia and attenuates brain injury after intracerebral hemorrhage. J. Cereb. Blood Flow Metab. 37:2383–2395. 10.1177/0271678X16666551 27596835 PMC5482387

[bib16] Li M., Li Z., Yao Y., Jin W.N., Wood K., Liu Q., Shi F.D., and Hao J. 2017 b Astrocyte-derived interleukin-15 exacerbates ischemic brain injury via propagation of cellular immunity. Proc. Natl. Acad. Sci. USA. 114:E396–E405. 10.1073/pnas.1612930114 27994144 PMC5255606

[bib17] Li M., Ren H., Sheth K.N., Shi F.D., and Liu Q. 2017 c A TSPO ligand attenuates brain injury after intracerebral hemorrhage. FASEB J. 31:3278–3287. 10.1096/fj.201601377RR 28416580 PMC5503714

[bib18] Liu Q., Jin W.N., Liu Y., Shi K., Sun H., Zhang F., Zhang C., Gonzales R.J., Sheth K.N., La Cava A., 2017 Brain Ischemia Suppresses Immunity in the Periphery and Brain via Different Neurogenic Innervations. Immunity. 46:474–487. 10.1016/j.immuni.2017.02.015 28314594

[bib19] Liu Q., Sanai N., Jin W.N., La Cava A., Van Kaer L., and Shi F.D. 2016 Neural stem cells sustain natural killer cells that dictate recovery from brain inflammation. Nat. Neurosci. 19:243–252. 10.1038/nn.4211 26752157 PMC5336309

[bib20] Lodoen M., Ogasawara K., Hamerman J.A., Arase H., Houchins J.P., Mocarski E.S., and Lanier L.L. 2003 NKG2D-mediated natural killer cell protection against cytomegalovirus is impaired by viral gp40 modulation of retinoic acid early inducible 1 gene molecules. J. Exp. Med. 197:1245–1253. 10.1084/jem.20021973 12756263 PMC2193789

[bib21] Long E.O., Kim H.S., Liu D., Peterson M.E., and Rajagopalan S. 2013 Controlling natural killer cell responses: integration of signals for activation and inhibition. Annu. Rev. Immunol. 31:227–258. 10.1146/annurev-immunol-020711-075005 23516982 PMC3868343

[bib22] Moxon-Emre I., and Schlichter L.C. 2011 Neutrophil depletion reduces blood-brain barrier breakdown, axon injury, and inflammation after intracerebral hemorrhage. J. Neuropathol. Exp. Neurol. 70:218–235. 10.1097/NEN.0b013e31820d94a5 21293296

[bib23] Mracsko E., and Veltkamp R. 2014 Neuroinflammation after intracerebral hemorrhage. Front. Cell. Neurosci. 8:388 10.3389/fncel.2014.00388 25477782 PMC4238323

[bib24] Mracsko E., Javidi E., Na S.Y., Kahn A., Liesz A., and Veltkamp R. 2014 Leukocyte invasion of the brain after experimental intracerebral hemorrhage in mice. Stroke. 45:2107–2114. 10.1161/STROKEAHA.114.005801 24916913

[bib25] Murthy S.B., Urday S., Beslow L.A., Dawson J., Lees K., Kimberly W.T., Iadecola C., Kamel H., Hanley D.F., Sheth K.N., ; VISTA ICH Collaborators 2016 Rate of perihaematomal oedema expansion is associated with poor clinical outcomes in intracerebral haemorrhage. J. Neurol. Neurosurg. Psychiatry. 87:1169–1173. 10.1136/jnnp-2016-313653 27466360 PMC5299159

[bib26] Qureshi A.I., Mendelow A.D., and Hanley D.F. 2009 Intracerebral haemorrhage. Lancet. 373:1632–1644. 10.1016/S0140-6736(09)60371-8 19427958 PMC3138486

[bib27] Ren H., Kong Y., Liu Z., Zang D., Yang X., Wood K., Li M., and Liu Q. 2018 Selective NLRP3 (Pyrin Domain-Containing Protein 3) Inflammasome Inhibitor Reduces Brain Injury After Intracerebral Hemorrhage. Stroke. 49:184–192. 10.1161/STROKEAHA.117.018904 29212744 PMC5753818

[bib28] Sansing L.H., Harris T.H., Kasner S.E., Hunter C.A., and Kariko K. 2011 Neutrophil depletion diminishes monocyte infiltration and improves functional outcome after experimental intracerebral hemorrhage. Acta Neurochir. Suppl. (Wien). 111:173–178. 10.1007/978-3-7091-0693-8_29 PMC3702167 21725751

[bib29] Selim M., Foster L.D., Moy C.S., Xi G., Hill M.D., Morgenstern L.B., Greenberg S.M., James M.L., Singh V., Clark W.M., ; i-DEF Investigators 2019 Deferoxamine mesylate in patients with intracerebral haemorrhage (i-DEF): a multicentre, randomised, placebo-controlled, double-blind phase 2 trial. Lancet Neurol. 18:428–438. 10.1016/S1474-4422(19)30069-9 30898550 PMC6494117

[bib30] Shi F.D., Ljunggren H.G., La Cava A., and Van Kaer L. 2011 Organ-specific features of natural killer cells. Nat. Rev. Immunol. 11:658–671. 10.1038/nri3065 21941294 PMC3620656

[bib31] Thiex R., and Tsirka S.E. 2007 Brain edema after intracerebral hemorrhage: mechanisms, treatment options, management strategies, and operative indications. Neurosurg. Focus. 22 E6 10.3171/foc.2007.22.5.7 17613237

[bib32] Urday S., Beslow L.A., Dai F., Zhang F., Battey T.W., Vashkevich A., Ayres A.M., Leasure A.C., Selim M.H., Simard J.M., 2016 Rate of Perihematomal Edema Expansion Predicts Outcome After Intracerebral Hemorrhage. Crit. Care Med. 44:790–797. 10.1097/CCM.0000000000001553 26757167 PMC4859217

[bib33] Urday S., Kimberly W.T., Beslow L.A., Vortmeyer A.O., Selim M.H., Rosand J., Simard J.M., and Sheth K.N. 2015 Targeting secondary injury in intracerebral haemorrhage--perihaematomal oedema. Nat. Rev. Neurol. 11:111–122. 10.1038/nrneurol.2014.264 25623787

[bib34] Vivier E., Raulet D.H., Moretta A., Caligiuri M.A., Zitvogel L., Lanier L.L., Yokoyama W.M., and Ugolini S. 2011 Innate or adaptive immunity? The example of natural killer cells. Science. 331:44–49. 10.1126/science.1198687 21212348 PMC3089969

[bib35] Wang J. 2010 Preclinical and clinical research on inflammation after intracerebral hemorrhage. Prog. Neurobiol. 92:463–477. 10.1016/j.pneurobio.2010.08.001 20713126 PMC2991407

[bib36] Zhang Y., Gao Z., Wang D., Zhang T., Sun B., Mu L., Wang J., Liu Y., Kong Q., Liu X., 2014 Accumulation of natural killer cells in ischemic brain tissues and the chemotactic effect of IP-10. J. Neuroinflammation. 11:79 10.1186/1742-2094-11-79 24742325 PMC4039314

[bib37] Zheng H., Chen C., Zhang J., and Hu Z. 2016 Mechanism and Therapy of Brain Edema after Intracerebral Hemorrhage. Cerebrovasc. Dis. 42:155–169. 10.1159/000445170 27110940

[bib38] Zhu J., Huang X., and Yang Y. 2010 NKG2D is required for NK cell activation and function in response to E1-deleted adenovirus. J. Immunol. 185:7480–7486. 10.4049/jimmunol.1002771 21076062 PMC3008345

