# Supplementary material for: Brain transforms natural killer cells that exacerbate brain edema after intracerebral hemorrhage
Source: J Exp Med. 2020 Sep 1;217(12):e20200213. doi: 10.1084/jem.20200213 (PMC7526480; doi:10.1084/jem.20200213)
Supplement: Table S1 — shows patient characteristics. [file JEM_20200213_TableS1.docx]

Table S1. Patient characteristics

| Subject | Sex | Age (yr) | Hematoma size (ml) | Location | Time of surgery (h after onset) | Sample type |
| --- | --- | --- | --- | --- | --- | --- |
| ICH 1 | F | 67 | 50 | Left basal ganglia | 5 | Blood and brain |
| ICH 2 | M | 47 | 55 | Parietal lobe | 7 | Blood and brain |
| ICH 3 | M | 57 | 70 | Left basal ganglia | 9 | Blood and brain |
| ICH 4 | M | 50 | 60 | Right basal ganglia | 5.5 | Blood and brain |
| ICH 5 | M | 59 | 63 | Left basal ganglia | 6 | Blood and brain |
| ICH 6 | M | 63 | 100 | Left basal ganglia | 12 | Blood and brain |
| ICH 7 | F | 61 | 70 | Left basal ganglia | 4.5 | Blood and brain |
| ICH 8 | M | 38 | 50 | Left basal ganglia | 6.5 | Blood and brain |
| ICH 9 | M | 80 | 60 | Left basal ganglia | 4.5 | Blood and brain |
| Control 1 | F | 68 |  |  |  | Brain |
| Control 2 | M | 37 |  |  |  | Brain |
| Control 3 | M | 65 |  |  |  | Brain |
| Control 4 | M | 37 |  |  |  | Brain |
| Control 5 | M | 66 |  |  |  | Brain |
| Control 6 | F | 48 |  |  |  | Brain |
| Control 7 | F | 57 |  |  |  | Brain |
| Control 8 | M | 72 |  |  |  | Brain |
| Control 9 | M | 44 |  |  |  | Brain |
| Control 10 | M | 58 |  |  |  | Blood |
| Control 11 | F | 46 |  |  |  | Blood |
| Control 12 | F | 70 |  |  |  | Blood |
| Control 13 | M | 63 |  |  |  | Blood |
| Control 14 | M | 68 |  |  |  | Blood |
| Control 15 | M | 58 |  |  |  | Blood |
| Control 16 | M | 53 |  |  |  | Blood |
| Control 17 | F | 56 |  |  |  | Blood |
| Control 18 | M | 63 |  |  |  | Blood |

F, female; M, male.
